# Supplementary material for: Amino Acid Variation in HLA Class II Proteins Is a Major Determinant of Humoral Response to Common Viruses
Source: Am J Hum Genet. 2015 Oct 8;97(5):738–43. doi: 10.1016/j.ajhg.2015.09.008 (PMC4667104; doi:10.1016/j.ajhg.2015.09.008)
Supplement: Document S1. Figures S1–S7 and Tables S1–S3, S6, S9, and S10 [file mmc1.pdf]

The American Journal of Human Genetics
Supplemental Data

Amino Acid Variation in HLA Class II Proteins Is a Major Determinant of Humoral Response to Common Viruses

Christian Hammer, Martin Begemann, Paul J. McLaren, István Bartha, Angelika Michel, Beate Klose, Corinna Schmitt, Tim Waterboer, Michael Pawlita, Thomas F. Schulz, Hannelore Ehrenreich, and Jacques Fellay

Supplemental Figures.

Figure S1. Principal component analysis.

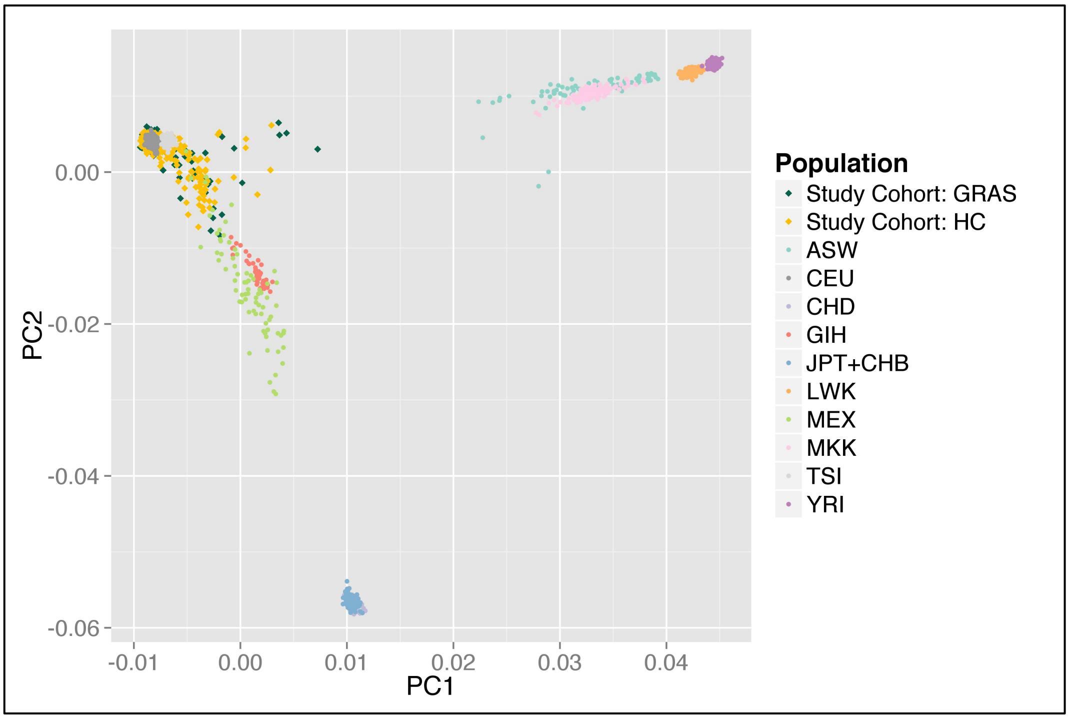

Study participants are displayed together with Hapmap version3 r2 samples, according to their first two principal components (PC1, PC2) as generated using GCTA (v1.24). GRAS, Göttingen Research Association for Schizophrenia patients; HC, Neuropsychiatrically healthy controls; ASW, African ancestry in Southwest USA; CEU, Utah residents with Northern and Western European ancestry from the CEPH collection; CHB, Han Chinese in Beijing, China; CHD, Chinese in Metropolitan Denver, Colorado; GIH, Gujarati Indians in Houston, Texas; JPT, Japanese in Tokyo, Japan; LWK, Luhya in Webuye, Kenya; MXL, Mexican Ancestry in Los Angeles, California; MKK, Maasai in Kinyawa, Kenya, TSI, Toscani in Italia; YRI, Yoruba in Ibadan, Nigeria.

Figure S2. Quantile-quantile plot for assessment of test statistic inflation.

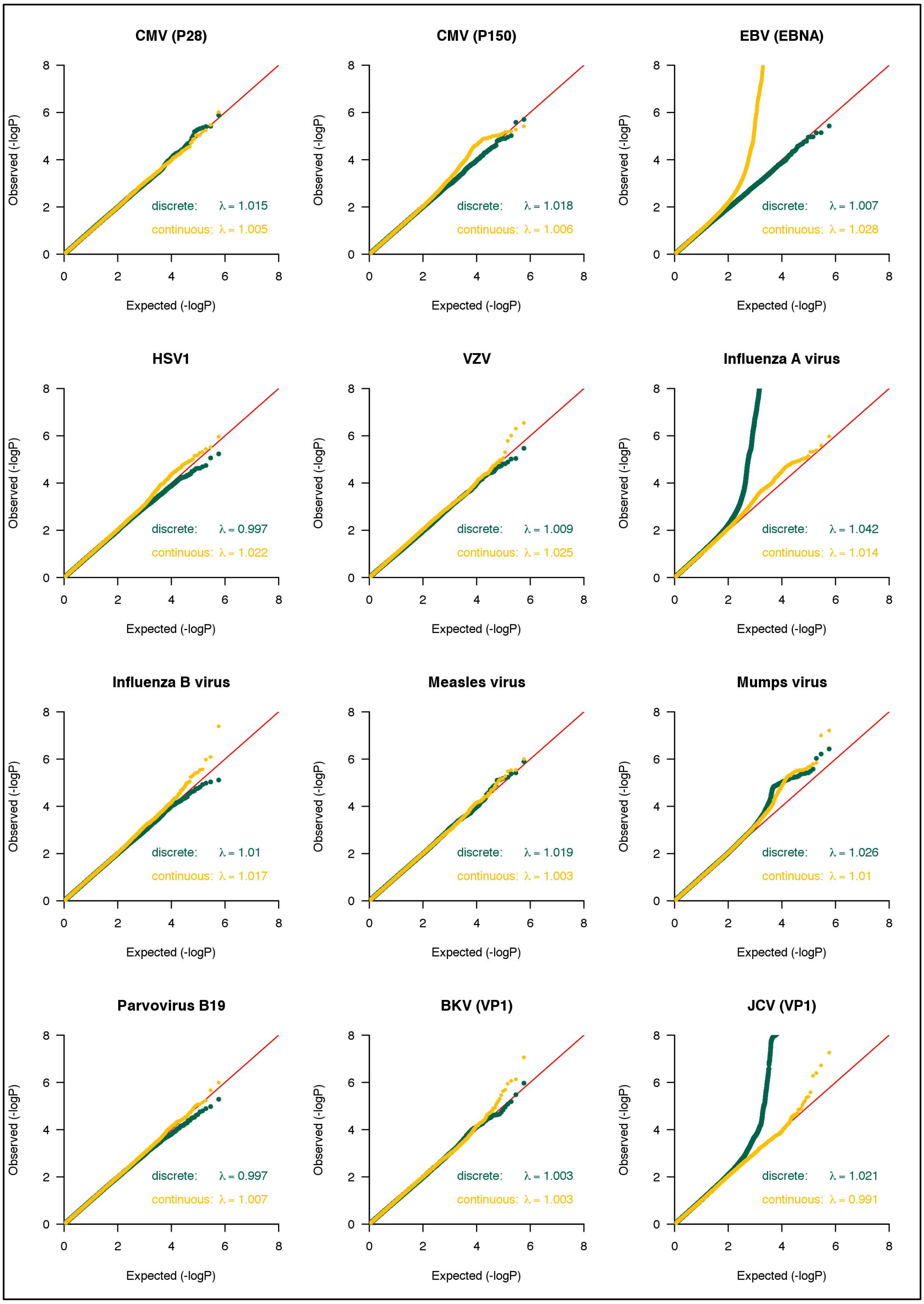

Continued on next page.

Figure S2. Quantile-quantile plot for assessment of test statistic inflation (continued).

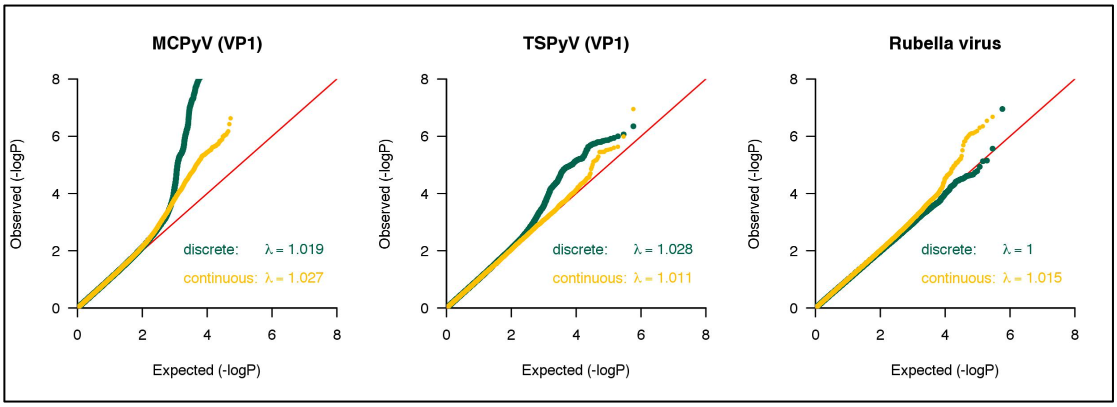

Quantile-quantile plot for each virus, including results for serostatus (discrete trait, green) and IgG levels (continuous trait, yellow). For each tested variant, the –log10 *P* value is plotted against the null distribution (red line). λ, genomic inflation factor.

Figure S3. Manhattan plots of association results.

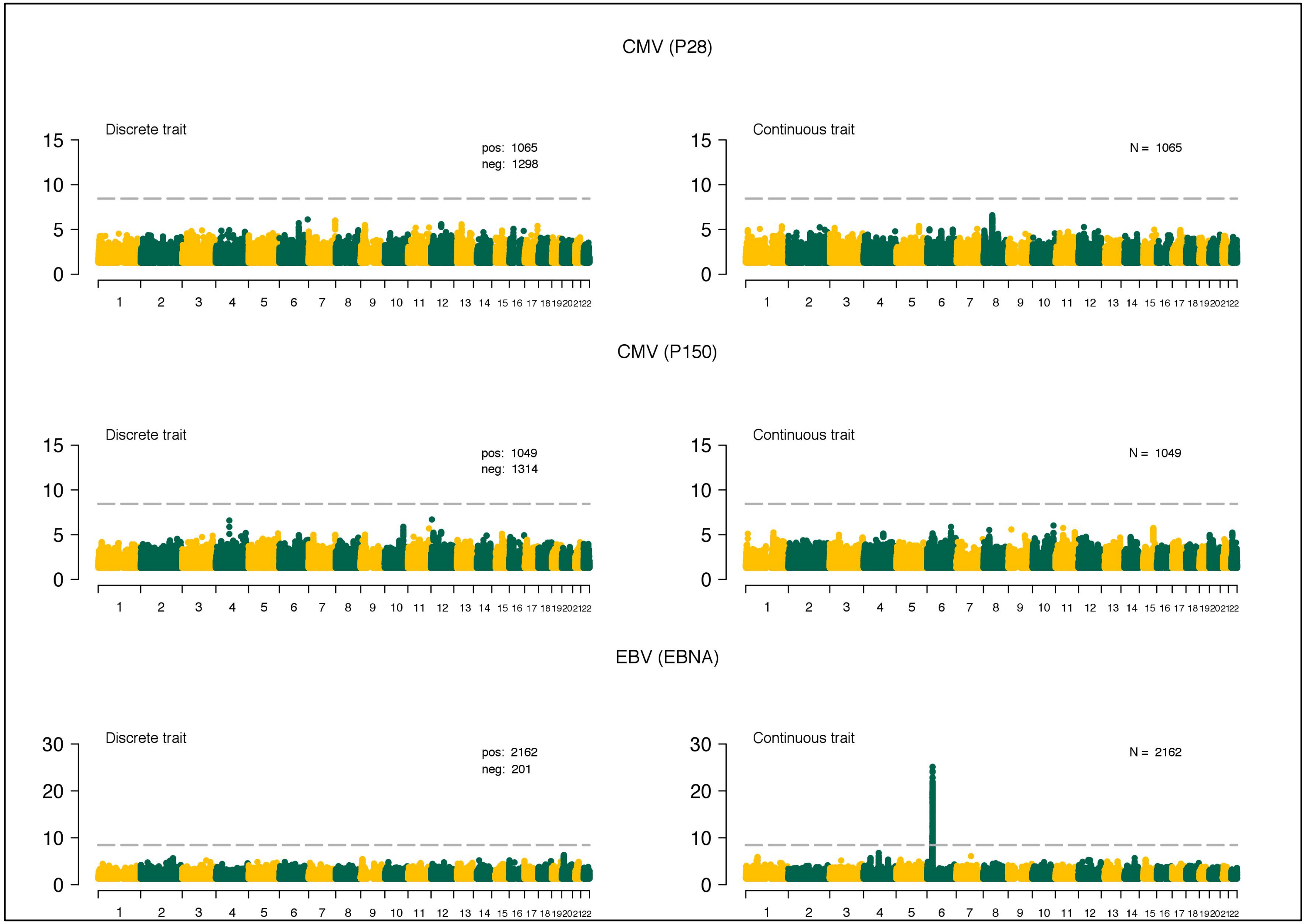

Continued on next page.

Figure S3. Manhattan plots of association results (continued).

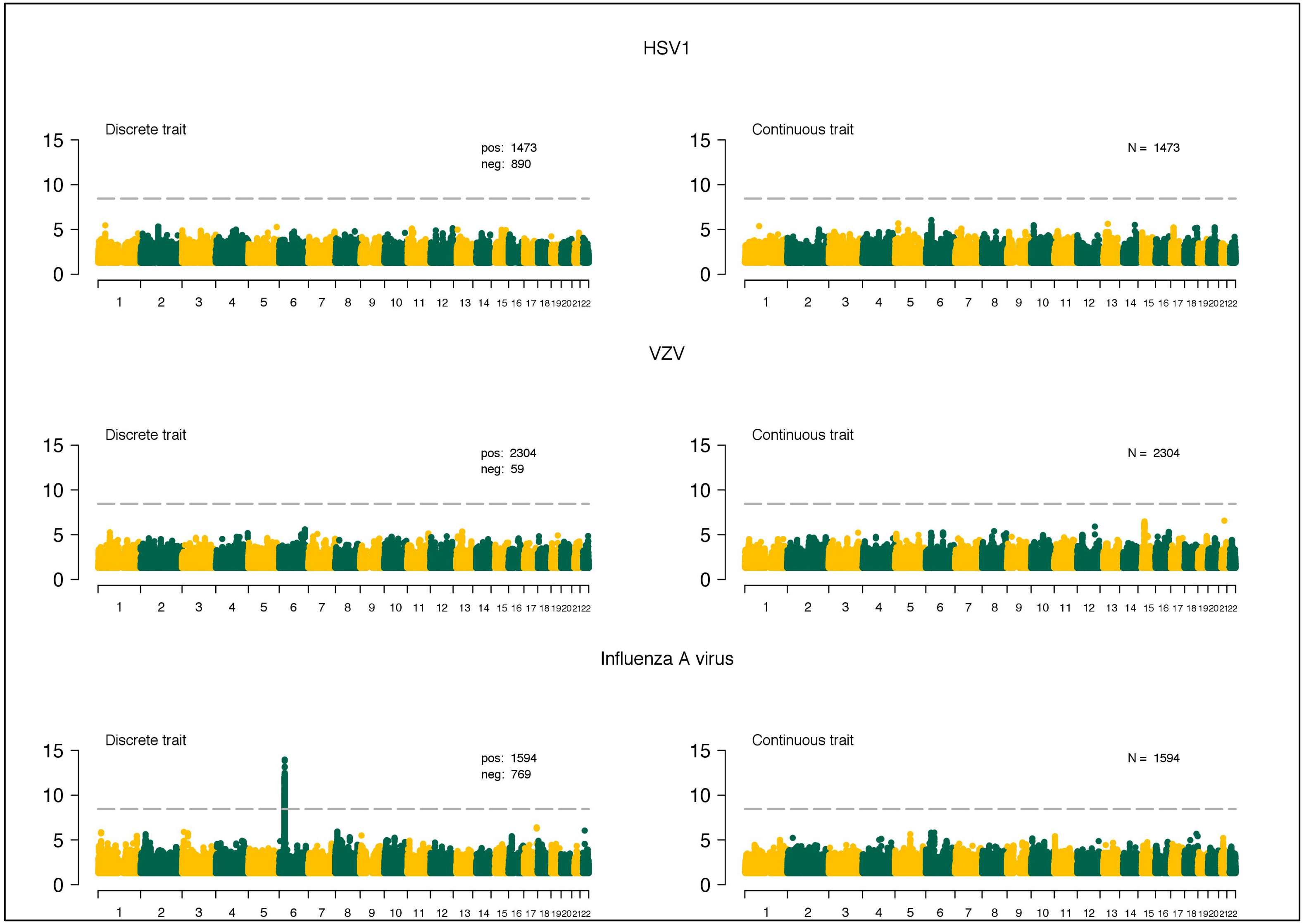

Continued on next page.

Figure S3. Manhattan plots of association results (continued).

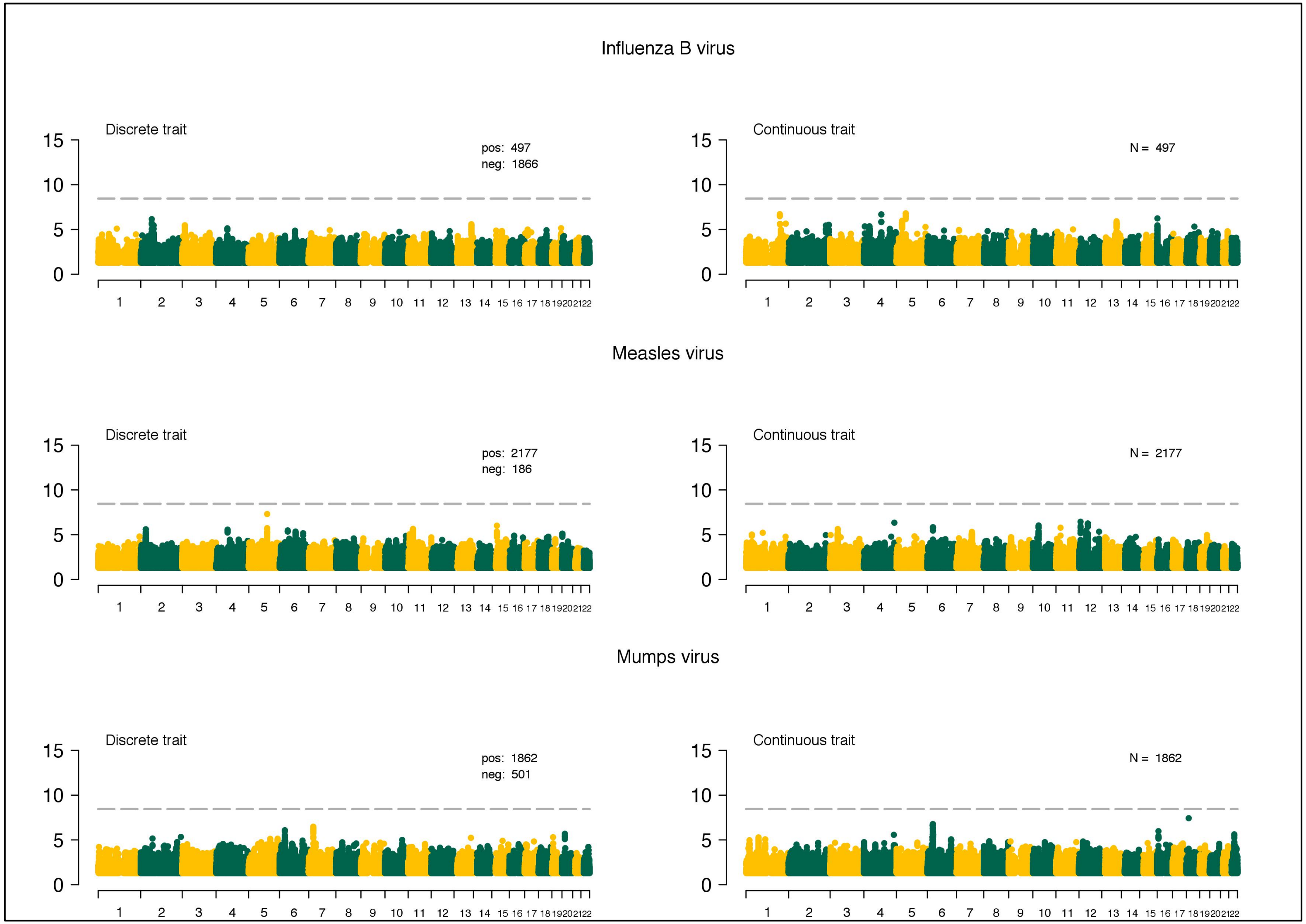

Continued on next page.

Figure S3. Manhattan plots of association results (continued).

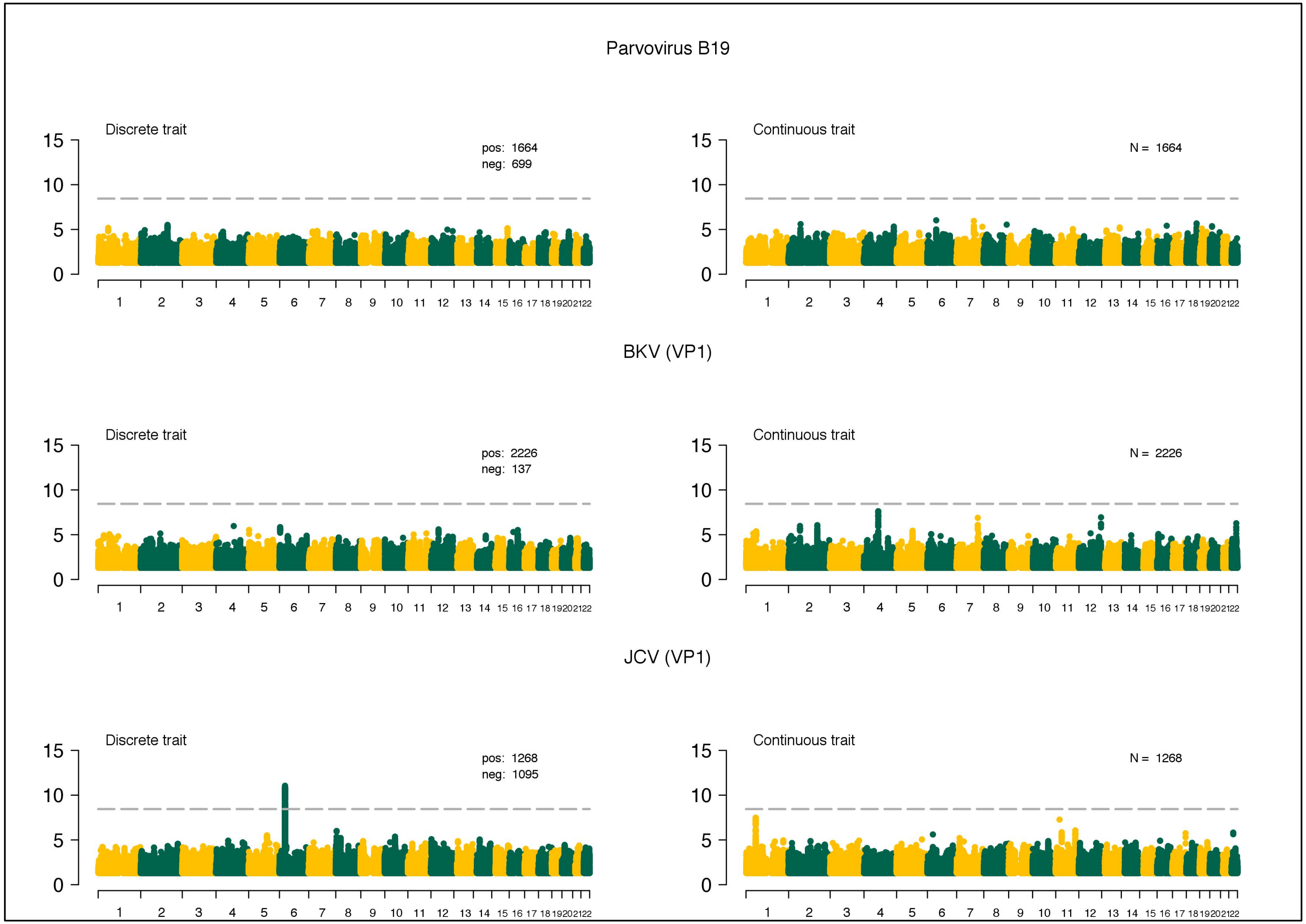

Continued on next page.

Figure S3. Manhattan plots of association results (continued).

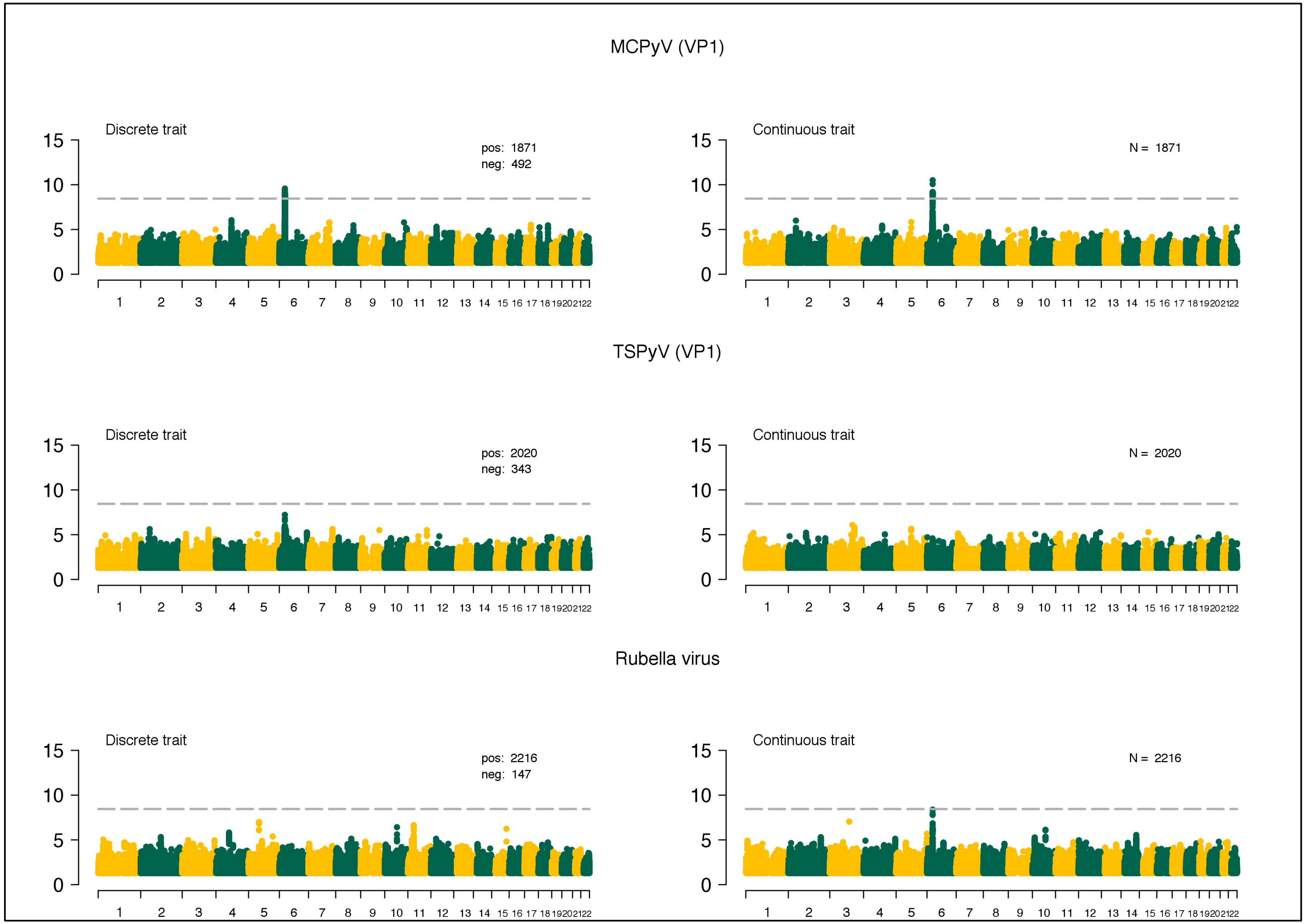

Manhattan plots of the association results for each tested virus, both for serostatus (discrete trait, left panel) and IgG levels (continuous trait, right panel). The number of included subjects is given for each analysis. Each variant is plotted by genomic position (x-axis) and -log10(*P*) value (y-axis). The dashed horizontal line denotes genome-wide significance (*P* = 3.57 x 10-9).

Figure S4. GWAS and HLA association results for EBV anti-EBNA IgG levels.

A

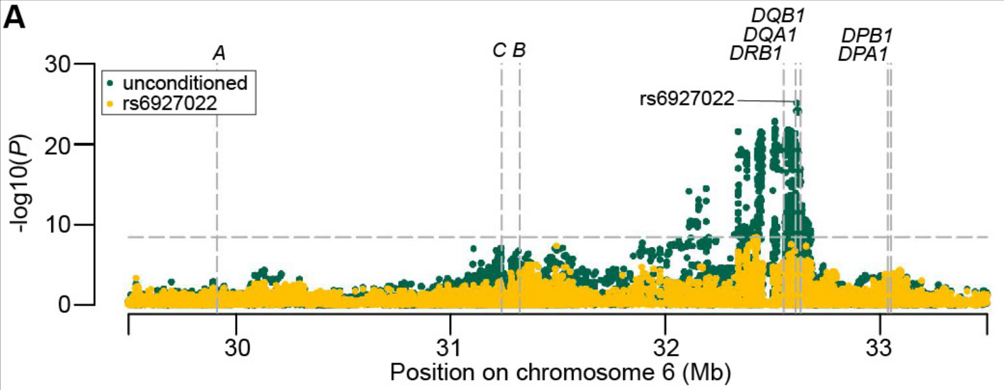

B

HLA-DRβ1 (11)	*HLA-DRB1* alleles	frequency	Beta (95% CI)
Leu	*01:01	11.1%	-0.02 (-0.06,0.03)
Ser	*03:01,*08:01,*11:01,*11:04,*12:01,*13:01, *13:02,*13:03,*14:01	42.9%	0.08 (0.06,0.11)
Val	*04:01,*04:04	15.7%	-0.10 (-0.14,-0.06)
Gly	*07:01	12.0%	-0.17 (-0.21,-0.12)
Pro	*15:01,*16:01	17.4%	0.07 (0.04,0.11)

HLA-DRβ1 (26)*	*HLA-DRB1* alleles	frequency	Beta (95% CI)
Leu	*01:01,*12:01	14.1%	0.10 (0.02,0.18)
Tyr	*03:01	11.6%	-0.16 (-0.21,-0.10)
Phe	*04:01,*04:04,*07:01,*08:01,*11:01,*11:04, *13:01,*13:02,*13:03,*14:01,*15:01,*16:01	74.3%	0.09 (0.04,0.13)

*, conditioning on HLA-DRβ1 (11)

C

Covariates	Risk allele	P	Beta (95% CI)
age, sex, PC1-3	*HLA-DRB1*07:01*	1.0×10^{-14}	-0.166 (-0.21,-0.12)
+ *HLA-DRB1*07:01*	*HLA-DRB1*03:01*	2.6×10^{-9}	-0.115 (-0.15,-0.08)
+ *HLA-DRB1*03:01*	*HLA-DRB1*04:04*	1.2×10^{-5}	-0.207 (-0.30,-0.12)

(A) Regional association plot of GWAS results for EBV anti-EBNA IgG levels of 2162 seropositive individuals. Values of –log10(*P*) are plotted by their position in the MHC genomic region on chromosome 6. The most significant association was observed for rs6927022 ($P = 7.3 \times 10^{-26}$, beta = 0.16 for the A allele). Accounting for the effect of rs6927022, we observed no further independent association. The dashed horizontal line indicates the threshold for genome-wide significance. The annotated dashed vertical lines indicate the positions of the classical HLA genes. (B) Effect estimates for amino acid residues at position 11 (omnibus $P = 5.9 \times 10^{-23}$) and 26 (conditional omnibus $P = 2.5 \times 10^{-9}$) of HLA-DRβ1. Designated classical HLA alleles contain the respective amino acid residue at the given position. CI, confidence interval. (C) Association results and conditional regression for classical HLA alleles. Conditioning on *HLA-DRB1*07:01* revealed *HLA-DRB1*03:01* to be independently associated. PC, principal component; CI, confidence interval.

Figure S5. GWAS and HLA association results for JCPyV serostatus.

A

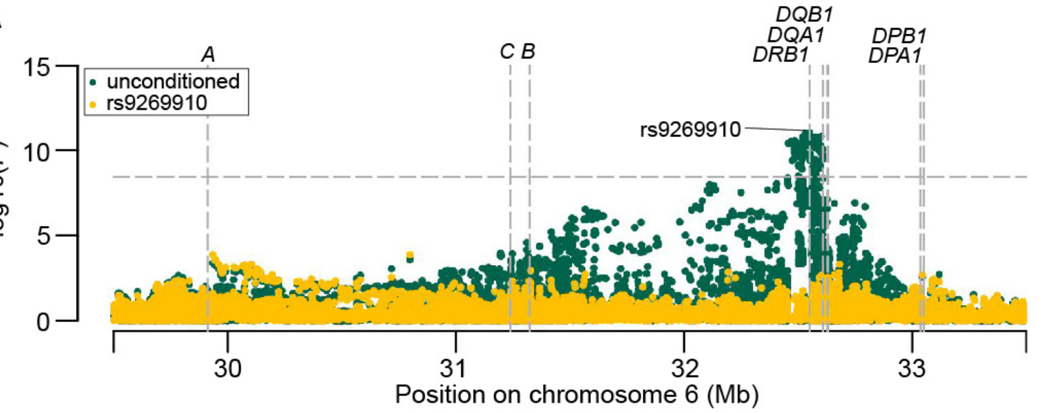

B

HLA-DRβ1 (133)	*HLA-DRB1* alleles	frequency	OR (95% CI)
Arg	*01:01,*03:01,*04:01,*04:04,*07:01,*08:01, *11:01,*11:03,*11:04,*12:01,*13:01,*13:02, *13:03,*14:01	82.6%	1.71 (1.46,2.01)
Leu	*15:01,*16:01	17.4%	0.58 (0.50,0.68)

C

Covariates	Risk allele	P	OR (95% CI)
age, sex, PC1-3, diagnosis	*HLA-DQA1*01:02*	4.1 x 10^{-9}	0.64 (0.56,0.75)
+ *HLA-DQA1*01:02*	*HLA-DRB1*04:04*	2.8 x 10^{-4}	0.46 (0.31,0.70)

(A) Regional association plot of GWAS results for JCPyV serostatus, comparing 1268 seropositive and 1095 seronegative individuals. Values of –log10(*P*) are plotted by their position in the MHC genomic region on chromosome 6. The most significant association was observed for rs9269910 (P = 8.9 x 10^{-12}, OR = 1.74 for the A allele). Accounting for the effect of rs9269910, we observed no further independent association. The dashed horizontal line indicates the threshold for genome-wide significance. The annotated dashed vertical lines indicate the positions of the classical HLA genes. (B) Effect estimates for amino acid residues at position 133 of HLA-DRβ1 (omnibus P = 1.1 x 10^{-11}). Designated classical HLA alleles contain the respective amino acid residue at the given position. OR, odds ratio; CI, confidence interval. (C) Association results and conditional regression for classical HLA alleles. Conditioning on *HLA-DQA1*01:02* did not reveal further independent associations (threshold: P = 3.57 x 10^{-09}). PC, principal component; OR, odds ratio; CI, confidence interval.

Figure S6. GWAS and HLA association results for MCPyV serostatus.

A

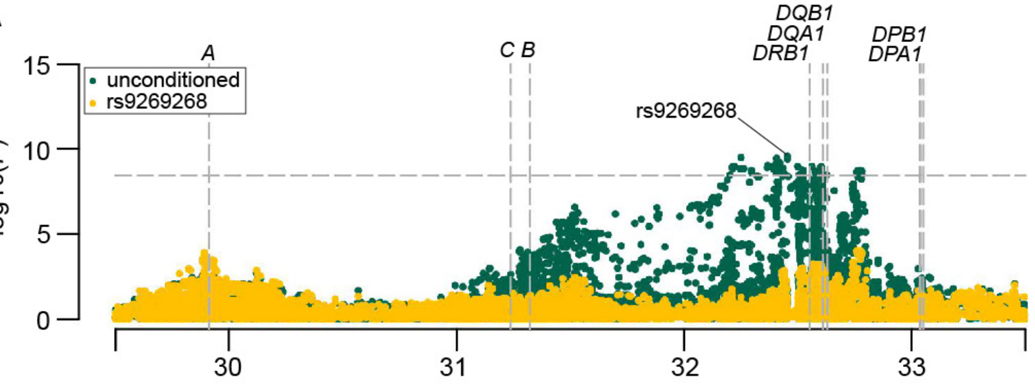

B

HLA-DRβ1 (13)	*HLA-DRB1* alleles	frequency	OR (95% CI)
Phe	*01:01	12.6%	1.42 (1.13,1.78)
Ser	*03:01,*11:01,*11:03,*11:04,*13:01,*13:02, *13:03,*14:01	37.7%	1.45 (1.24,1.69)
His	*04:01,*04:04	15.0%	0.81 (0.67,0.97)
Tyr	*07:01	12.0%	0.98 (0.79,1.21)
Gly	*08:01,*12:01	5.3%	0.89 (0.66,1.20)
Arg	*15:01,*16:01	17.4%	0.60 (0.50,0.71)

C

Covariates	Risk allele	P	OR (95% CI)
age, sex, PC1-3	*HLA-DQB1*06:02*	1.4×10^{-9}	0.56 (0.46,0.67)
+ *HLA-DQB1*06:02*	*HLA-DQA1*03:01*	1.0×10^{-3}	0.73 (0.60,0.88)

(A) Regional association plot of GWAS results for MCPyV serostatus, comparing 1871 seropositive and 492 seronegative individuals. Values of –log10(*P*) are plotted by their position in the MHC genomic region on chromosome 6. The most significant association was observed for rs9269268 ($P = 2.7 \times 10^{-10}$, OR = 1.53 for the C allele). Accounting for the effect of rs9269268, we observed no further independent association. The dashed horizontal line indicates the threshold for genome-wide significance. The annotated dashed vertical lines indicate the positions of the classical HLA genes. (B) Effect estimates for amino acid residues at position 13 of HLA-DRβ1 (omnibus $P = 1.9 \times 10^{-10}$). Designated classical HLA alleles contain the respective amino acid residue at the given position. OR, odds ratio; CI, confidence interval. (C) Association results and conditional regression for classical HLA alleles. Conditioning on *HLA-DQB1*06:02* did not reveal further independent associations (threshold: P = 3.57×10^{-09}). PC, principal component; OR, odds ratio; CI, confidence interval.

Figure S7. GWAS association results for MCPyV IgG levels.

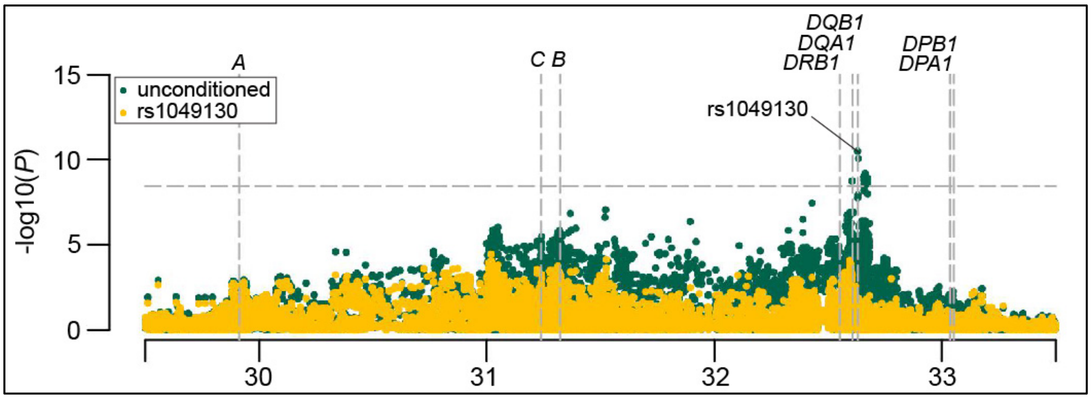

Regional association plot of GWAS results for MCPyV IgG levels of 1871 seropositive individuals. Values of –log10(*P*) are plotted by their position in the MHC genomic region on chromosome 6. The most significant association was observed for rs1049130 (P = 3.2 x 10^{-11}, beta = 0.22 for the G allele). Accounting for the effect of rs1049130, we observed no further independent association. The dashed horizontal line indicates the threshold for genome-wide significance. The annotated dashed vertical lines indicate the positions of the classical HLA genes.

Supplemental Tables.

Table S1. Description of serological assays.

Virus	Test / Epitope	Details of Antigens for Serology Testing
**CMV**[1]	P28	Strain “Town” (FJ616285), small nucleocapsid protein p28
	P150	Strain “Town” (FJ616285), N-terminal part of tegument protein p150, amino acids 1-550
**EBV**[1; 2]	EBNA	Strain “B-95-8” (P03211.1), truncated protein: C-terminal part, amino acids 325-641
**HSV1**	Novagnost HSV1 IgG	Commercially available purified recombinant glycoprotein G, expressed in eukaryotic cells (Siemens Novagnost)
**VZV**	Enzygnost Anti-VZV / IgG	Commercially available antigen, strain “Ellen”, prepared from infected human diploid lung fibroblasts (Siemens Enzygnost)
**Influenza A**	Novagnost Influenza A IgG	Commercially available cell-free H3N2 virus preparation, cultured in chicken eggs (Siemens Novagnost)
**Influenza B**	Novagnost Influenza B IgG	Commercially available Influenza B strain, cultured in eukaryotic cells (Siemens Novagnost)
**Measles**	Enzygnost Anti-Measles Virus / IgG	Commercially available antigen, strain “Edmonston”, prepared from infected Vero cells (Siemens Enzygnost)
**Mumps**	Enzygnost Anti-Parotitis Virus / IgG	Commercially available antigen, strain “Enders”, prepared from infected human Vero cells (Siemens Enzygnost)
**Parvo B19**	Novagnost Parvovirus B 19 IgG	Commercially available purified recombinant VLP antigen, expressed in yeast (Siemens Novagnost)
**BKPyV**[3; 4]	VP1	Strain “AS” (P14996.1), major capsid protein VP1
**JCPyV**[3; 4]	VP1	Strain “GS/B” (NC_001699.1), major capsid protein VP1
**MCPyV**[5; 6]	VP1	Isolate “344” (JF812999.1), major capsid protein VP1
**TSPyV**[7]	VP1	(NC_014361.1), major capsid protein VP1
**Rubella**	Enzygnost Anti-Rubella Virus / IgG	Commercially available antigen, strain RA27/3, substrain B1272, prepared from infected BHK cells (Siemens Enzygnost)

NCBI accession numbers are given in brackets, where available.

Table S2. Impact of age and sex on serostatus and IgG levels.

	Age				Sex			
	Serostatus		IgG levels		Serostatus		IgG levels	
Virus	*P* (logistic regression)	OR (95% CI)	*P* (linear regression)	Beta (95% CI)	*P* (logistic regression)	OR (reference = male) (95% CI)	*P* (linear regression)	Beta (reference = male) (95% CI)
**BKPyV**	**3.0E-03**	0.982 (0.970,0.994)	**1.9E-26**	-0.016 (-0.019,-0.013)	5.9E-01	0.907 (0.636,1.292)	8.4E-01	-0.009 (-0.093,0.076)
**CMV PP150**	**2.0E-16**	1.026 (1.020,1.033)	7.0E-03	0.006 (0.002,0.011)	1.2E-01	1.141 (0.964,1.350)	**2.3E-07**	0.339 (0.211,0.466)
**CMV PP28**	**2.9E-12**	1.022 (1.016,1.028)	8.0E-03	0.006 (0.002,0.011)	4.8E-02	1.185 (1.002,1.402)	**2.0E-03**	0.211 (0.077,0.346)
**EBV**	**1.2E-09**	1.039 (1.026,1.052)	7.0E-01	0.000 (-0.002,0.001)	5.0E-03	1.584 (1.148,2.186)	8.5E-02	-0.036 (-0.076,0.005)
**HSV1**	**1.3E-30**	1.041 (1.034-1.048)	4.5E-02	0.002 (0.000-0.003)	1.9E-01	1.125 (0.945,1.338)	5.8E-02	-0.040 (-0.082,0.001)
**Influenza A**	2.3E-01	0.996 (0.990,1.002)	**5.4E-05**	0.003 (0.001,0.004)	4.7E-01	1.068 (0.892,1.277)	2.6E-01	0.028 (-0.017,0.062)
**Influenza B**	**2.0E-17**	1.032 (1.025,1.040)	9.0E-03	-0.002 (-0.003,0.000)	4.1E-02	0.804 (0.652,0.991)	9.9E-02	-0.027 (-0.060,0.005)
**JCPyV**	**1.6E-10**	1.020 (1.014,1.027)	3.5E-01	0.002 (-0.002,0.005)	3.2E-02	0.832 (0.703,0.984)	1.5E-02	-0.117 (-0.211,-0.022)
**MCPyV**	**1.7E-04**	1.015 (1.007,1.023)	**1.2E-04**	0.007 (0.003,0.010)	1.1E-01	0.846 (0.690,1.037)	7.8E-01	-0.014 (-0.111,0.083)
**Measles**	**2.5E-22**	1.084 (1.066,1.102)	**1.1E-111**	0.031 (0.029,0.034)	2.1E-01	1.227 (0.892,1.689)	9.0E-03	0.106 (0.026,0.186)
**Mumps**	**1.0E-19**	1.039 (1.031,1.048)	**3.5E-04**	0.004 (0.002,0.007)	3.7E-02	1.249 (1.013,1.540)	**4.1E-04**	0.123 (0.055,0.192)
**Parvo**	2.6E-01	0.996 (0.990,1.003)	**4.8E-12**	-0.005 (-0.006,-0.004)	7.0E-03	0.780 (0.650,0.935)	**3.0E-06**	0.093 (0.054,0.132)
**Rubella**	**6.0E-10**	1.049 (1.033,1.065)	1.5E-01	-0.002 (-0.005,0.001)	**5.6E-08**	3.677 (2.298,5.882)	**9.0E-07**	-0.188 (-0.262,-0.113)
**TSPyV**	**3.1E-08**	1.026 (1.017,1.036)	**1.7E-07**	-0.010 (-0.013,-0.006)	1.2E-01	0.832 (0.658,1.051)	**2.0E-03**	-0.160 (-0.262,-0.059)
**VZV**	1.1E-01	1.017 (0.996,1.037)	1.1E-01	-0.002 (-0.003,0.000)	4.9E-01	1.217 (0.700,2.115)	4.1E-01	0.022 (-0.031,0.076)

Significant *P* values (corrected for the number of viruses) are displayed in bold. OR, odds ratio; CI, confidence interval.

Table S3. Comparison of serostatus and IgG levels between psychiatric disease patients and healthy control individuals.

Virus	Serostatus				IgG levels			
	N seropositive (%)		*P* (logistic regression)	OR (95% CI)	Mean IgG (SE)		*P* linear regression)	Beta (95% CI)
	Healthy controls	Psychiatric diagnosis			Healthy controls	Psychiatric diagnosis		
**BKPyV**	1090 (95.0)	1136 (93.4)	1.4E-01	0.76 (0.53,1.09)	8.40 (0.03)	8.49 (0.03)	**2.1E-04**	0.15 (0.07,0.23)
**CMV PP150**	518 (45.2)	531 (43.7)	**2.0E-03**	0.76 (0.64,0.90)	7.72 (0.04)	7.99 (0.05)	**2.0E-04**	0.24 (0.11,0.36)
**CMV PP28**	522 (45.5)	543 (44.7)	1.2E-02	0.80 (0.68,0.95)	7.90 (0.05)	8.04 (0.05)	1.8E-01	0.09 (-0.04,0.22)
**EBV**	1071 (93.4)	1091 (89.7)	**4.9E-05**	0.53 (0.39,0.72)	8.86 (0.01)	8.92 (0.01)	1.0E-02	0.05 (0.01,0.09)
**HSV1**	699 (60.9)	774 (63.7)	5.4E-01	0.95 (0.79,1.13)	10.35 (0.01)	10.42 (0.01)	**2.0E-03**	0.07 (0.03,0.11)
**Influenza A**	774 (67.5)	820 (67.4)	8.0E-01	1.02 (0.86,1.22)	9.97 (0.01)	9.94 (0.01)	5.2E-02	-0.04 (-0.08,0.00)
**Influenza B**	236 (20.6)	261 (21.5)	5.0E-01	0.93 (0.76,1.15)	9.51 (0.01)	9.55 (0.01)	**1.0E-03**	0.05 (0.02,0.08)
**JCPyV**	546 (47.6)	722 (59.4)	**7.0E-06**	1.47 (1.24,1.73)	7.11 (0.03)	7.17 (0.03)	2.7E-01	0.05 (-0.04,0.14)
**MCPyV**	893 (77.9)	978 (80.4)	6.7E-01	1.05 (0.85,1.28)	7.97 (0.04)	8.04 (0.03)	4.5E-01	0.04 (-0.06,0.13)
**Measles**	1044 (91.0)	1133 (93.2)	9.1E-01	1.02 (0.74,1.40)	14.99 (0.03)	15.14 (0.03)	1.0E-01	0.06 (-0.01,0.13)
**Mumps**	902 (78.6)	960 (78.9)	3.0E-01	0.90 (0.73,1.10)	14.53 (0.02)	14.62 (0.02)	2.6E-02	0.08 (0.01,0.14)
**Parvo**	849 (74.0)	815 (67.0)	**2.2E-04**	0.71 (0.59,0.85)	10.91 (0.01)	10.85 (0.01)	2.0E-02	-0.04 (-0.08.-0.01)
**Rubella**	1089 (94.9)	1127 (92.7)	6.0E-03	0.61 (0.43,0.87)	11.41 (0.03)	11.41 (0.03)	8.4E-01	-0.01 (-0.08,0.07)
**TSPyV**	973 (84.8)	1047 (86.1)	9.4E-01	0.99 (0.78,1.26)	8.91 (0.03)	8.95 (0.04)	6.8E-02	0.09 (-0.01,0.19)
**VZV**	1128 (98.3)	1176 (96.7)	7.0E-03	0.46 (0.26,0.81)	13.72 (0.02)	13.78 (0.02)	7.0E-03	0.07 (0.02,0.12)

Age, sex, and the first three principal components are included as covariates in all regression analyses. Significant *P* values (corrected for the number of viruses) are displayed in bold. OR, odds ratio; CI, confidence interval; SE, standard error of the mean.

Table S6. Residual significance of GWAS SNPs conditioning for the associated HLA alleles and variable amino acids.

Virus	Phenotype	Covariates	SNP	*P* (SNP)	Associated classical HLA alleles	*P* (SNP) conditional on significant HLA alleles
**EBV**	IgG levels	age,sex,PC1-3	rs6927022	7.35×10^{-26}	DRB1*07:01, DRB1*03:01	2.59×10^{-11}
**Influenza A**	Serostatus	age,sex,PC1-3	rs140012631	1.06×10^{-14}	DQB1*05:01	4.47×10^{-4}
**JCPyV**	Serostatus	age,sex,PC1-3,diagnosis	rs9269910	8.88×10^{-12}	DQA1*01:02	3.63×10^{-4}
**MCPyV**	Serostatus	age,sex,PC1-3	rs9269268	2.67×10^{-10}	DQB1*06:02	2.74×10^{-2}
**Virus**	**Phenotype**	**Covariates**	**SNP**	***P* (SNP)**	**Associated HLA- DRβ1 amino acid positions**	***P* (SNP) conditional on significant amino acids**
**EBV**	IgG levels	age,sex,PC1-3	rs6927022	7.35×10^{-26}	11, 26	4.20×10^{-2}
**Influenza A**	Serostatus	age,sex,PC1-3	rs140012631	1.06×10^{-14}	96	9.48×10^{-4}
**JCPyV**	Serostatus	age,sex,PC1-3,diagnosis	rs9269910	8.88×10^{-12}	133	1
**MCPyV**	Serostatus	age,sex,PC1-3	rs9269268	2.67×10^{-10}	13	4.34×10^{-2}

PC, principal component.

Table S9. Variance explained by the associated variable HLA amino acids.

Study Design	Virus	Nagelkerke's pseudo-r^2 (age, sex, PC1-3)	associated amino acid positions	Nagelkerke's pseudo-r^2 (age, sex, PC1-3, amino acids)	Δr^2
Discrete	**Influenza A**	0.4%	HLA-DRβ1 (96)	4.8%	4.4%
	**JCPyV**[a]	4.7%	HLA-DRβ1 (133)	7.2%	2.5%
	**MCPyV**	2.4%	HLA-DRβ1 (13)	5.9%	3.5%
**Study Design**	**Virus**	**r^2 (age, sex, PC1-3)**	**associated amino acid positions**	**r^2 (age, sex, PC1-3, amino acids)**	**Δr^2**
**Continuous**	**EBV**	1.2%	HLA-DRβ1 (11,26)	6.6%	5.4%

[a],additionally corrected for diagnosis (psychiatric patients vs. healthy controls)

Table S10. Correlations of effect estimates between analyses of serostatus and IgG levels.

Virus	N seropositive (%)	Variable HLA amino acid positions	
		**Pearson's r**	***P***
**EBV**	2162 (91.5)	0.39	< 2.2E-16
**Influenza A**	1594 (67.5)	0.62	< 2.2E-16
**JCPyV**	1268 (53.7)	0.66	< 2.2E-16
**MCPyV**	1871 (79.2)	0.55	< 2.2E-16

Correlation analyses between odds ratios of logistic regression analyses on serostatus and betas of linear regression analyses on IgG levels, taking into account all variable amino acid positions. r, correlation coefficient

Supplemental References.

1. Brozy, J. (2009). Development of Multiplex Serology for HSV-1, HSV-2, EBV and HCMV using Recombinant Proteins. Ruprecht-Karls-Universität Heidelberg, Heidelberg.
2. Teras, L.R., Rollison, D.E., Pawlita, M., Michel, A., Brozy, J., de Sanjose, S., Blase, J.L., and Gapstur, S.M. (2015). Epstein-Barr virus and risk of non-Hodgkin lymphoma in the cancer prevention study-II and a meta-analysis of serologic studies. International journal of cancer Journal international du cancer 136, 108-116.
3. Antonsson, A., Green, A.C., Mallitt, K.A., O'Rourke, P.K., Pawlita, M., Waterboer, T., and Neale, R.E. (2010). Prevalence and stability of antibodies to the BK and JC polyomaviruses: a long-term longitudinal study of Australians. The Journal of general virology 91, 1849-1853.
4. Kjaerheim, K., Roe, O.D., Waterboer, T., Sehr, P., Rizk, R., Dai, H.Y., Sandeck, H., Larsson, E., Andersen, A., Boffetta, P., et al. (2007). Absence of SV40 antibodies or DNA fragments in prediagnostic mesothelioma serum samples. International journal of cancer Journal international du cancer 120, 2459-2465.
5. Robles, C., Casabonne, D., Benavente, Y., Costas, L., Gonzalez-Barca, E., Aymerich, M., Campo, E., Tardon, A., Jimenez-Moleon, J.J., Castano-Vinyals, G., et al. (2015). Seroreactivity against Merkel cell polyomavirus and other polyomaviruses in chronic lymphocytic leukemia, the MCC-Spain study. The Journal of general virology.
6. Rollison, D.E., Giuliano, A.R., Messina, J.L., Fenske, N.A., Cherpelis, B.S., Sondak, V.K., Roetzheim, R.G., Iannacone, M.R., Michael, K.M., Gheit, T., et al. (2012). Case-control study of Merkel cell polyomavirus infection and cutaneous squamous cell carcinoma. Cancer epidemiology, biomarkers & prevention : a publication of the American Association for Cancer Research, cosponsored by the American Society of Preventive Oncology 21, 74-81.
7. Teras, L.R., Rollison, D.E., Pawlita, M., Michel, A., Blase, J.L., Willhauck-Fleckenstein, M., and Gapstur, S.M. (2015). Prediagnostic circulating polyomavirus antibody levels and risk of non-Hodgkin lymphoma. Cancer epidemiology, biomarkers & prevention : a publication of the American Association for Cancer Research, cosponsored by the American Society of Preventive Oncology 24, 477-480.
