# Supplementary material for: Amino Acid Variation in HLA Class II Proteins Is a Major Determinant of Humoral Response to Common Viruses
Source: Am J Hum Genet. 2015 Oct 8;97(5):738–43. doi: 10.1016/j.ajhg.2015.09.008 (PMC4667104; doi:10.1016/j.ajhg.2015.09.008)
Supplement: Document S2. Article plus Supplemental Data [file mmc6.pdf]

Amino Acid Variation in HLA Class II Proteins Is a Major Determinant of Humoral Response to Common Viruses

Christian Hammer,[1,2,3,*] Martin Begemann,[3] Paul J. McLaren,[1,2] István Bartha,[1,2] Angelika Michel,[4] Beate Klose,[5] Corinna Schmitt,[5] Tim Waterboer,[4] Michael Pawlita,[4] Thomas F. Schulz,[5] Hannelore Ehrenreich,[3,6,7] and Jacques Fellay[1,2,7]

The magnitude of the human antibody response to viral antigens is highly variable. To explore the human genetic contribution to this variability, we performed genome-wide association studies of the immunoglobulin G response to 14 pathogenic viruses in 2,363 immunocompetent adults. Significant associations were observed in the major histocompatibility complex region on chromosome 6 for influenza A virus, Epstein-Barr virus, JC polyomavirus, and Merkel cell polyomavirus. Using local imputation and fine mapping, we identified specific amino acid residues in human leucocyte antigen (HLA) class II proteins as the most probable causal variants underlying these association signals. Common HLA-DRβ1 haplotypes showed virus-specific patterns of humoral-response regulation. We observed an overlap between variants affecting the humoral response to influenza A and EBV and variants previously associated with autoimmune diseases related to these viruses. The results of this study emphasize the central and pathogen-specific role of HLA class II variation in the modulation of humoral immune response to viral antigens in humans.

The humoral immune response plays an essential role in the control and prevention of viral infections in humans. It has long been known that concentrations of serum immunoglobulins vary from person to person,[1] and antibody titers against prevalent viruses have been shown to be highly variable in the population.[2,3] A significant fraction of that variation is heritable,[1,3] yet little is known about the human genetic control and regulation of the immunoglobulin response to specific pathogens.

To investigate the impact of common human genetic variation on humoral immunity and to identify pathogen-specific variants associated with antibody response, we measured serum immunoglobulin G (IgG) levels against 14 common viruses (Table 1) in 2,363 immunocompetent adults of European ancestry (Figure S1) with available genome-wide genotype data,[4] comprising 1,147 anonymized blood donors (62.0% male, mean age ± SD = 37.5 ± 13.2) and 1,216 individuals with psychiatric diagnoses (64.9% male, mean age ± SD = 40.6 ± 13.5) who were recruited for the Göttingen Research Association for Schizophrenia (GRAS).[5,6] All study participants provided informed consent, including consent for genetic testing, and the GRAS data collection has been approved by the ethical committee of the Georg-August-Universität Göttingen as well as by the respective local regulatories and ethical committees of all collaborating centers.[6] All subject data were collected in accordance with ethical guidelines and the Helsinki Declaration.[7]

A list and description of all assays used for determination of IgG levels is provided in Table S1. We used multiplex serology on the Luminex platform, based on glutathione S-transferase (GST) fusion capture immunosorbent assays combined with fluorescent-bead technology,[8] or commercially available ELISA-based Enzygnost or Novagnost assays (Siemens Healthcare Diagnostics). The latter were automatically processed on the BEP III System (Siemens Healthcare Diagnostics) and interpreted (according to the manufacturer's instructions) as positive, negative, or borderline (the latter of was defined as negative for the purpose of statistics). For the Luminex-based assays, seronegativity was defined as the absence of detectable IgG.

Genome-wide SNP genotyping was performed with an Axiom myDesign genotyping array (Affymetrix) and was subject to stringent quality control steps as described previously.[4] Imputation of unobserved genotypes was performed with the 1000 Genomes Project phase 1 v.3 haplotypes as a reference panel. Genotypes were prephased with MaCH v.1[9] and subsequently imputed by Minimac.[10] SNPs with a reported r^2 quality metric score < 0.8 or a minor allele frequency (MAF) $< 5\%$, as well as reported markers on sex chromosomes, were excluded from downstream analyses. SNPs were also filtered on the basis of "missingness" (excluded if they had $< 95\%$ genotyping rate) and marked deviation from the Hardy-Weinberg equilibrium (excluded if $p < 5 \times 10^{-7}$). We then used logistic or linear regression models

[1]School of Life Sciences, École Polytechnique Fédérale de Lausanne, 1015 Lausanne, Switzerland; [2]Swiss Institute of Bioinformatics, 1015 Lausanne, Switzerland; [3]Clinical Neuroscience, Max Planck Institute of Experimental Medicine, 37075 Göttingen, Germany; [4]Division of Molecular Diagnostics of Oncogenic Infections, Infections and Cancer Program, German Cancer Research Center, 69120 Heidelberg, Germany; [5]Institute of Virology, Hannover Medical School, 30625 Hannover, Germany; [6]DFG Research Center for Nanoscale Microscopy and Molecular Physiology of the Brain, 37075 Göttingen, Germany
[7]These authors contributed equally to this work
*Correspondence: christian.hammer@epfl.ch
http://dx.doi.org/10.1016/j.ajhg.2015.09.008. ©2015 The Authors
This is an open access article under the CC BY license (http://creativecommons.org/licenses/by/4.0/).

Table 1. List of Analyzed Viruses and Seroprevalence

Family	Species	Name of Test or Epitope	N Seropositive (%)	IgG Levels[a]
Herpesviridae	cytomegalovirus	P28	1,065 (45.1)	8.0 ± 1.1
		P150	1,049 (44.4)	7.9 ± 1.0
	Epstein-Barr virus	EBNA	2,162 (91.5)	8.9 ± 0.5
	herpes simplex virus-1	Novagnost HSV1 IgG	1,473 (62.3)	10.4 ± 0.4
	varizella zoster virus	Enzygnost Anti-VZV/IgG	2,304 (97.5)	13.7 ± 0.6
Orthomyxoviridae	influenza A virus	Novagnost Influenza A IgG	1,594 (67.5)	10.0 ± 0.4
	influenza B virus	Novagnost Influenza B IgG	497 (21.0)	9.5 ± 0.2
Paramyxoviridae	measles virus	Enzygnost Anti-Measles Virus/IgG	2,177 (92.1)	15.1 ± 0.9
	mumps virus	Enzygnost Anti-Parotitis Virus/IgG	1,862 (78.8)	14.6 ± 0.7
Parvoviridae	parvovirus B19	Novagnost Parvovirus B 19 IgG	1,664 (70.4)	10.9 ± 0.4
Polyomaviridae	BK polyomavirus	VP1	2,226 (94.2)	8.4 ± 1.0
	JC polyomavirus	VP1	1,268 (53.7)	7.1 ± 0.8
	Merkel cell polyomavirus	VP1	1,871 (79.2)	8.0 ± 1.0
	trichodysplasia spinulosa-associated polyomavirus	VP1	2,020 (85.5)	8.9 ± 1.1
Togaviridae	rubella virus	Enzygnost Anti-Rubella Virus/IgG	2,216 (93.8)	11.4 ± 0.9

[a]Log normalized, mean ± SD.

in PLINK v.1.9[11] to test for association between ~six million SNPs and IgG response to 14 common human viruses (Table 1), using both a case-control study design (serostatus: antibody positive versus negative) and a continuous, quantitative approach (log-normalized IgG levels in seropositive samples). The first three principal components, calculated with GCTA (v.1.24),[12] as well as sex and age, which affect humoral response phenotypes (Table S2), were included as covariates in all analyses. In case of significant differences in serostatus or IgG levels between healthy control individuals and individuals affected by neuropsychiatric disease (Table S3), psychiatric diagnosis was included as an additional binary covariate. We observed no evidence of residual inflation in any test statistic ($\lambda = 0.99$–1.04, Figure S2).

Correcting for the number of SNPs and viruses tested, we observed genome-wide significant signals ($p < 3.57 \times 10^{-9}$) in the human leucocyte antigen (HLA) class II region of the major histocompatibility complex (MHC) on chromosome 6 for influenza A virus, Epstein-Barr virus (EBV), JC polyomavirus (JCPyV), and Merkel cell polyomavirus (MCPyV) (Table 2, Figure S3). Full summary association results are available for download from Zenodo.

To fine map the associated region and pinpoint potentially functional variants, we imputed four-digit classical HLA alleles and variable amino acid positions in the HLA

Table 2. Summary of Genome-Wide Significant Association Results

		Study Design: Serostatus			Study Design: IgG Levels
		Influenza A	JCPyV	MCPyV	EBV
SNP	ID (coded allele)	rs140012631 (C)	rs9269910 (A)	rs9269268 (C)	rs6927022 (A)
	p value	1.06×10^{-14}	8.88×10^{-12}	2.67×10^{-10}	7.35×10^{-26}
	OR or beta[a] (95% CI)	2.02 (1.84,2.19)	1.74 (1.58,1.90)	1.53 (1.40,1.66)	0.16 (0.13,0.19)
Classical HLA allele	Allele	*HLA-DQB1*05:01*	*HLA-DQA1*01:02*	*HLA-DQB1*06:02*	*HLA-DRB1*07:01*
	p value	4.91×10^{-12}	4.11×10^{-9}	1.35×10^{-9}	1.01×10^{-14}
	OR or beta[a] (95% CI)	0.53 (0.44,0.63)	0.65 (0.56,0.75)	0.56 (0.46,0.67)	−0.17 (−0.21,−0.12)
Amino acid	Protein (position)	HLA-DRβ1 (96)	HLA-DRβ1 (133)	HLA-DRβ1 (13)	HLA-DRβ1 (11)
	Omnibus p value	2.64×10^{-16}	1.27×10^{-11}	1.87×10^{-10}	5.85×10^{-23}

The strongest associated GWAS SNP, classical HLA allele, and amino acid position for each genome-wide significant virus is shown, along with corresponding p values and effect estimates. OR, odds ratio; CI, confidence interval.
[a]OR is shown for the serostatus study design, and beta is shown for the IgG levels study design.

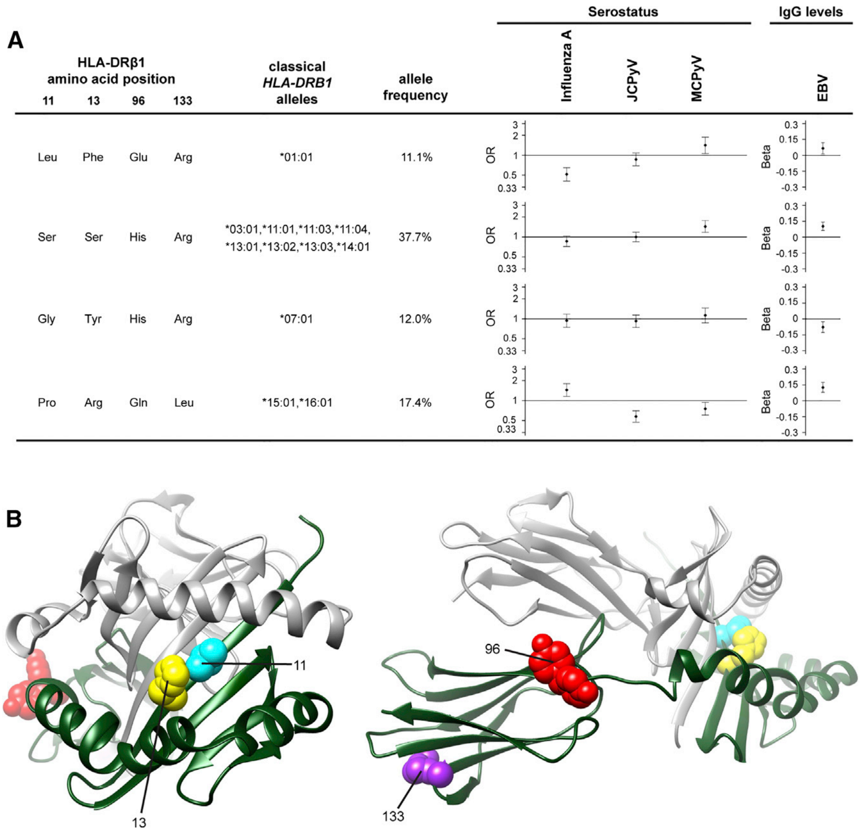

Figure 1. Effect of Common HLA-DRβ1 Haplotypes on Virus Serostatus or Antibody Levels

(A) Estimated effects for HLA-DRβ1 haplotypes as defined by the strongest associated amino acid positions for influenza A virus, JCPyV, and MCPyV serostatus, as well as EBV IgG levels (Table 2). These four positions were imputed with SNP2HLA and all showed an imputation accuracy of > 99% in the original publication.[13] The Val-His-Tyr-Arg-encoding haplotype with a frequency of 14.9%, present in classical HLA alleles *HLA-DRB1*04:01* and *HLA-DRB1*04:04*, was chosen as reference (i.e., given an OR of 1, or a beta of 0, respectively) and not included in the figure. Only common haplotypes with a frequency of > 10% were included in the analysis and accounted for 93.1% of haplotype diversity. Diamonds designate estimated effect sizes; error bars define the 95% confidence interval. OR, odds ratio

(B) 3D model of the HLA-DR αβ heterodimer. The protein is shown in front (left) and side (right) views. The DR α chain is displayed in gray and the β chain in green. Associated amino acid positions, as selected for haplotype analysis, are highlighted. This figure was prepared with UCSF chimera,[14] with Protein Data Bank code PDB: 4MCY.[15]

class I and II proteins by using SNP2HLA and the T1DGC Immunochip/HLA reference panel[13] and tested these for association with IgG response (Tables S4 and S5). 101 HLA alleles and 200 amino acids had a MAF > 1% and were included in the analysis (r^2 quality metric score: median = 0.99, interquartile range = 0.98–1), and we used a multi-degree-of-freedom omnibus test to test for association at multi-allelic amino acid positions with three or more possible states. For all viruses with genome-wide significant SNPs, we found corresponding associations with HLA class II alleles and amino acids (Table 2), and they explained most of the SNP association signals when included as covariates in conditional analyses (Table S6). The residual SNP significance observed in some of the conditional analyses suggests the existence of additional associated alleles or amino acids that did not reach genome-wide significance, although non-classical MHC associations or HLA imputation errors could also play a role. Among imputed amino acids, the strongest associations were observed for positions in HLA-DRβ1 (Table 2, Table S5). We therefore analyzed the potential impact of common HLA-DRβ1 haplotypes on IgG response to influenza A, EBV, JCPyV, and MCPyV (Figure 1). This analysis showed no consistency in the associations: the size and directionality of the effects were virus specific (Figure 1A). Most prominently, the amino acid haplotypes present in the classical alleles *HLA-DRB1*15:01* or *HLA-DRB1*16:01* were associated with influenza A seropositivity and higher anti-EBV IgG levels, but with JCPyV and MCPyV seronegativity. In contrast, the haplotype present in *HLA-DRB1*01:01* were associated with MCPyV seropositivity and higher anti-EBV IgG levels, but with influenza A seronegativity.

We next explored the virus-specific associations in more detail. Anti-influenza A IgG antibodies were detected in approximately two thirds of study participants (Table 1), and two SNPs mapping to the *HLA-DRB1* (MIM: 142857), *DQA1* (MIM: 146880), and *DQB1* (MIM: 604305) gene regions were identified as independently associated with influenza A seropositivity in the genome-wide SNP screen (Figure 2A). Amino acid position 96 in HLA-DRβ1 was associated more strongly with influenza A serostatus than any SNP or classical HLA allele (omnibus $p = 2.6 \times 10^{-16}$, Table 2) and explained most of the association signals of both independently associated SNPs when included in a conditional analysis. The residual significance of $p = 9.5 \times 10^{-4}$ for rs140012631 and $p = 1.0 \times 10^{-4}$ for rs9269912 might, however, imply that these SNPs tag additional classical or non-classical HLA effects that did not reach our significance threshold. Individuals carrying a glutamine at position 96 were more likely to have detectable levels of anti-influenza A IgG (odds ratio [OR] = 1.68), whereas the presence of a glutamic acid was associated with seronegativity (OR = 0.51, Figure 2B). These two amino acids are carried by the *HLA-DRB1*15:01* and the *HLA-DRB1*01:01* alleles, respectively. They are in almost perfect linkage disequilibrium (LD) with *HLA-DQB1*06:02* and *HLA-DQB1*05:01*, respectively (Table S7), which

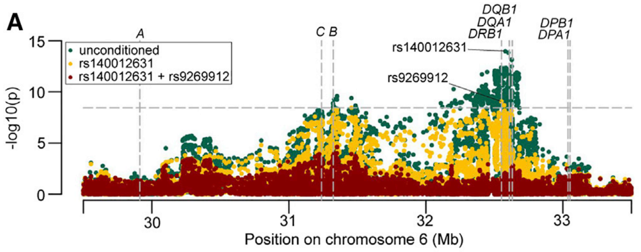

B

HLA-DRβ1 (96)	*HLA-DRB1* alleles	frequency	OR (95% CI)
Glu	***01:01**,*01:03	11.1%	0.51 (0.42,0.61)
His	*03:01,*07:01,*08:01,*11:01,*11:03*11:04, *12:01,*13:01,*13:02,*13:03,*14:01	55.9%	0.95 (0.84,1.08)
Tyr	*04:01,*04:04	14.9%	1.09 (0.92,1.30)
Gln	***15:01**,*16:01	18.1%	1.68 (1.41,2.00)

C

Covariates	Risk allele	p value	OR (95% CI)
age, sex, PC1-3	*HLA-DQB1*05:01*	4.9×10^{-12}	0.53 (0.44,0.63)
+ HLA-DQB1*05:01	*HLA-DQB1*06:02*	2.4×10^{-08}	1.78 (1.45,2.18)

Figure 2. GWAS and HLA association results for influenza A serostatus

(A) Regional association plot of GWAS results. Values of –log10(p) are plotted by their position in the MHC genomic region on chromosome 6. The most significant association was observed for rs140012631 ($p = 1.1 \times 10^{-14}$, OR = 2.01 for the C allele). Accounting for the effect of rs140012631, we observed an independent association at rs9269912 ($p = 8.5 \times 10^{-10}$, OR = 0.57 for the T allele). A further step of forward conditioning left no genome-wide significant signal. The dashed horizontal line indicates the threshold for genome-wide significance ($p = 3.57 \times 10^{-9}$). The annotated dashed vertical lines indicate the positions of the classical HLA genes.

(B) Effect estimates for amino acid residues at position 96 of HLA-DRβ1 (omnibus $p = 2.6 \times 10^{-16}$). Designated classical HLA alleles contain the respective amino acid residue at the given position. OR, odds ratio; CI, confidence interval.

(C) Association results and conditional regression for classical HLA alleles. Conditioning on *HLA-DQB1*05:01* did not reveal further independent associations (threshold: $p = 3.57 \times 10^{-9}$). PC, principal component; OR, odds ratio; CI, confidence interval.

were the most strongly associated classical HLA alleles (Figure 2C). This result is consistent with a recent study associating *HLA-DRB1*15:01* with increased responsiveness to trivalent influenza vaccines in Hispanic children.[16] Interestingly, the *HLA-DRB1*15:01-HLA-DQB1*06:02* haplotype is found in nearly 100% of individuals with narcolepsy (MIM: 161400),[17,18] a condition with strong evidence for an autoimmune basis and with a possible, but still controversial, mechanistic link to influenza infection.[19,20] The same HLA class II haplotype has also been shown to be positively associated with multiple sclerosis (MIM: 126200),[21] systemic lupus erythematosus (MIM: 152700),[22,23] and Goodpasture disease (MIM: 233450).[24]

More than 90% of study participants had detectable IgG against the EBV-encoded nuclear antigen-1 (EBNA-1), an antigen expressed in both latent and lytic modes of infection and that is essential for efficient EBV genome replication, persistence, and transcription in dividing cells. A large number of HLA SNPs were significantly associated with anti-EBNA-1 IgG levels (Figure S4A), consistent with the results of a previous study.[25] Among amino acids, positions 11 and 26 of HLA-DRβ1 showed strong, independent associations (Table 2, Figure S4B), which together explained the top SNP association from the genome-wide association studies (GWASs) (conditional p = 0.04, Table S6). Among classical alleles, *HLA-DRB1*07:01* showed the strongest association result (Figure S4C), and there was an independent association of *HLA-DRB1*03:01*. However, these two alleles could not completely explain the top GWAS hit (residual $p = 2.6 \times 10^{-11}$, Table S6), pointing to a possible relevance of additional classical HLA alleles that did not reach the significance threshold in our study. EBV is known or suspected to play a role in multiple sclerosis[26] and systemic autoimmune diseases,[27] and we observed an overlap between the genetic determinants of IgG response to EBV and the HLA class II variants that have been reported to associate with autoimmunity. Carriage of a glycine at HLA-DRβ1 position 11 was associated with decreased antibody levels (Figure S4B) and a lower risk of seropositive[28] and seronegative[29] rheumatoid arthritis (MIM: 180300). On the contrary, IgG levels were higher in individuals carrying a proline at position 11 (Figure S4B), which is in strong LD with alanine at position 71 ($r^2 = 0.83$, Table S8) and carried by the *HLA-DRB1*15:01* classical allele. HLA-DRβ1 position 71 and *HLA-DRB1*15:01* have been reported as the strongest genetic risk factors for multiple sclerosis at the level of amino acids and classical HLA alleles, respectively.[21] However, the directionality of immune response and autoimmune disease risk associations is not always consistent, and larger study cohorts will be required to determine the role and extent of the contribution of EBV immune response to autoimmune disease susceptibility.

We also observed significant SNP associations in the HLA class II region for JCPyV and MCPyV serostatus and for MCPyV IgG levels (Table 2, Figures S5A, S6A, and S7). HLA-DRβ1 positions 133 and 13 showed the strongest amino acid associations with JCPyV and MCPyV serostatus, respectively (Figures S5B and S6B), whereas *HLA-DQA1*01:02* and *HLA-DQB1*06:02* were associated with JCPyV and MCPyV seronegativity, respectively (Figures S5C and S6C). These two alleles are in strong LD with *HLA-DRB1*15:01* (Table S7), which was found to be associated with JCPyV seronegativity previously.[30] No classical HLA alleles or amino acids reached the significance threshold for MCPyV IgG levels.

Genome-wide significant HLA class II amino acids explained between 2.5% (JCPyV serostatus) and 5.4% (EBV anti-EBNA-1 IgG levels) of the phenotypic variance (Table S9). With our sample size, we had > 80% power to detect variants with a MAF of 10% that (1) explain at least 2.5% of the trait variance (IgG levels) in the case of 80% seropositivity or (2) have an OR of 1.5 for a discrete trait with 67% seropositivity.[31]

Although the continuous phenotype—IgG levels in seropositive individuals—is a direct indicator of the intensity of the humoral immune response, the interpretation

of the dichotomous phenotype—serostatus—is not as straightforward. Seronegativity can indeed reflect both non-exposure and IgG levels under the detection limit. A correlation analysis of ORs of the dichotomous trait and the respective betas of the continuous trait showed highly significant results (Table S10), suggesting that seronegativity results from very low IgG levels rather than from lack of exposure in a subset of the study population. Thus, we sought to maximize power by using the largest possible sample (i.e., by including seronegative individuals in the dichotomous analyses), particularly for viruses with a large seronegative fraction.

In summary, we here present a cross-pathogen, genome-wide investigation of the role of host genetics in modulating the individual IgG response to common viral antigens. We identified a strong association between HLA class II variation, in particular HLA-DRβ1 amino acid variants, and IgG response to several prevalent viruses. Our study illustrates the value of GWASs in infectious disease research and emphasizes the importance of fine mapping SNP association signals in the MHC via imputation of potentially functional HLA variation; doing so offers the unique possibility for a straightforward investigation of putative causal variants.[13] The pathogen-specific patterns of association that we observed suggest that small differences in the capacity of HLA-DRβ1 proteins to bind specific viral peptides and present them efficiently to CD4+ T cells can have a measurable impact on downstream antibody production. The concordance in associated genetic loci between IgG response to several common viral antigens and antibody-dependent autoimmune diseases observed in this study is intriguing. Although our results do not allow for the conclusion of a direct causative role of these viruses in autoimmune pathogenesis, they strongly encourage further genetic and functional work. Understanding how genetic differences affect human response to infection might ultimately inform drug and vaccine development and facilitate personalized preventive and therapeutic approaches.

Supplemental Data

Supplemental Data include seven figures and ten tables and can be found with this article online at http://dx.doi.org/10.1016/j.ajhg.2015.09.008.

Acknowledgments

We thank the study participants for their important contributions to this study. This work was supported by the Max Planck Society, the Max Planck Förderstiftung, and the DFG Research Center for Nanoscale Microscopy and Molecular Physiology of the Brain, as well as by the Niedersachsen-Research Network on Neuroinfectiology of the Ministry of Science and Culture of Lower Saxony. The computations were performed at the Vital-IT (http://www.vital-it.ch) center for high-performance computing of the SIB Swiss Institute of Bioinformatics. This work was also supported by a Young Investigator Grant from the Brain & Behavior Research Foundation, as well as a postdoctoral scholarship from the Daimler and Benz Foundation, both awarded to C.H. J.F. is supported by a professorship grant from the Swiss National Science Foundation (PP00P3_133703).

Received: June 26, 2015
Accepted: September 21, 2015
Published: October 8, 2015

Web Resources

The URLs for data presented herein are as follows:

1000 Genomes, http://browser.1000genomes.org
GCTA, http://www.complextraitgenomics.com/software/gcta/
Genetic Power Calculator, http://pngu.mgh.harvard.edu/~purcell/gpc/
MACH, http://www.sph.umich.edu/csg/abecasis/MACH/
Minimac, http://genome.sph.umich.edu/wiki/Minimac
OMIM, http://www.omim.org/
PLINK, https://www.cog-genomics.org/plink2
SNP2HLA, https://www.broadinstitute.org/mpg/snp2hla/
Zenodo, https://zenodo.org/record/18049

References

1. Grundbacher, F.J. (1974). Heritability estimates and genetic and environmental correlations for the human immunoglobulins G, M, and A. Am. J. Hum. Genet. *26*, 1–12.
2. Tsang, J.S., Schwartzberg, P.L., Kotliarov, Y., Biancotto, A., Xie, Z., Germain, R.N., Wang, E., Olnes, M.J., Narayanan, M., Golding, H., et al.; Baylor HIPC Center; CHI Consortium (2014). Global analyses of human immune variation reveal baseline predictors of postvaccination responses. Cell *157*, 499–513.
3. Rubicz, R., Leach, C.T., Kraig, E., Dhurandhar, N.V., Duggirala, R., Blangero, J., Yolken, R., and Göring, H.H. (2011). Genetic factors influence serological measures of common infections. Hum. Hered. *72*, 133–141.
4. Hammer, C., Stepniak, B., Schneider, A., Papiol, S., Tantra, M., Begemann, M., Sirén, A.L., Pardo, L.A., Sperling, S., Mohd Jofrry, S., et al. (2014). Neuropsychiatric disease relevance of circulating anti-NMDA receptor autoantibodies depends on blood-brain barrier integrity. Mol. Psychiatry *19*, 1143–1149.
5. Begemann, M., Grube, S., Papiol, S., Malzahn, D., Krampe, H., Ribbe, K., Friedrichs, H., Radyushkin, K.A., El-Kordi, A., Benseler, F., et al. (2010). Modification of cognitive performance in schizophrenia by complexin 2 gene polymorphisms. Arch. Gen. Psychiatry *67*, 879–888.
6. Ribbe, K., Friedrichs, H., Begemann, M., Grube, S., Papiol, S., Kästner, A., Gerchen, M.F., Ackermann, V., Tarami, A., Treitz, A., et al. (2010). The cross-sectional GRAS sample: a comprehensive phenotypical data collection of schizophrenic patients. BMC Psychiatry *10*, 91.
7. World Medical Association (2013). World Medical Association Declaration of Helsinki: ethical principles for medical research involving human subjects. JAMA *310*, 2191–2194.
8. Waterboer, T., Sehr, P., Michael, K.M., Franceschi, S., Nieland, J.D., Joos, T.O., Templin, M.F., and Pawlita, M. (2005). Multiplex human papillomavirus serology based on in situ-purified glutathione s-transferase fusion proteins. Clin. Chem. *51*, 1845–1853.
9. Li, Y., Willer, C.J., Ding, J., Scheet, P., and Abecasis, G.R. (2010). MaCH: using sequence and genotype data to estimate

haplotypes and unobserved genotypes. Genet. Epidemiol. *34*, 816–834.

10. Howie, B., Fuchsberger, C., Stephens, M., Marchini, J., and Abecasis, G.R. (2012). Fast and accurate genotype imputation in genome-wide association studies through pre-phasing. Nat. Genet. *44*, 955–959.
11. Chang, C.C., Chow, C.C., Tellier, L.C., Vattikuti, S., Purcell, S.M., and Lee, J.J. (2015). Second-generation PLINK: rising to the challenge of larger and richer datasets. Gigascience *4*, 7.
12. Yang, J., Lee, S.H., Goddard, M.E., and Visscher, P.M. (2011). GCTA: a tool for genome-wide complex trait analysis. Am. J. Hum. Genet. *88*, 76–82.
13. Jia, X., Han, B., Onengut-Gumuscu, S., Chen, W.M., Concannon, P.J., Rich, S.S., Raychaudhuri, S., and de Bakker, P.I. (2013). Imputing amino acid polymorphisms in human leukocyte antigens. PLoS ONE *8*, e64683.
14. Pettersen, E.F., Goddard, T.D., Huang, C.C., Couch, G.S., Greenblatt, D.M., Meng, E.C., and Ferrin, T.E. (2004). UCSF Chimera–a visualization system for exploratory research and analysis. J. Comput. Chem. *25*, 1605–1612.
15. Scally, S.W., Petersen, J., Law, S.C., Dudek, N.L., Nel, H.J., Loh, K.L., Wijeyewickrema, L.C., Eckle, S.B., van Heemst, J., Pike, R.N., et al. (2013). A molecular basis for the association of the HLA-DRB1 locus, citrullination, and rheumatoid arthritis. J. Exp. Med. *210*, 2569–2582.
16. Narwaney, K.J., Glanz, J.M., Norris, J.M., Fingerlin, T.E., Hokanson, J.E., Rewers, M., and Hambidge, S.J. (2013). Association of HLA class II genes with clinical hyporesponsiveness to trivalent inactivated influenza vaccine in children. Vaccine *31*, 1123–1128.
17. Mignot, E., Lin, L., Rogers, W., Honda, Y., Qiu, X., Lin, X., Okun, M., Hohjoh, H., Miki, T., Hsu, S., et al. (2001). Complex HLA-DR and -DQ interactions confer risk of narcolepsy-cataplexy in three ethnic groups. Am. J. Hum. Genet. *68*, 686–699.
18. Tafti, M., Hor, H., Dauvilliers, Y., Lammers, G.J., Overeem, S., Mayer, G., Javidi, S., Iranzo, A., Santamaria, J., Peraita-Adrados, R., et al. (2014). DQB1 locus alone explains most of the risk and protection in narcolepsy with cataplexy in Europe. Sleep *37*, 19–25.
19. Singh, A.K., Mahlios, J., and Mignot, E. (2013). Genetic association, seasonal infections and autoimmune basis of narcolepsy. J. Autoimmun. *43*, 26–31.
20. Partinen, M., Kornum, B.R., Plazzi, G., Jennum, P., Julkunen, I., and Vaarala, O. (2014). Does autoreactivity have a role in narcolepsy? Lancet Neurol. *13*, 1072–1073.
21. Patsopoulos, N.A., Barcellos, L.F., Hintzen, R.Q., Schaefer, C., van Duijn, C.M., Noble, J.A., Raj, T., Gourraud, P.A., Stranger, B.E., Oksenberg, J., et al.; IMSGC; ANZgene (2013). Fine-mapping the genetic association of the major histocompatibility complex in multiple sclerosis: HLA and non-HLA effects. PLoS Genet. *9*, e1003926.
22. Dong, R.P., Kimura, A., Hashimoto, H., Akizuki, M., Nishimura, Y., and Sasazuki, T. (1993). Difference in HLA-linked genetic background between mixed connective tissue disease and systemic lupus erythematosus. Tissue Antigens *41*, 20–25.
23. Hong, G.H., Kim, H.Y., Takeuchi, F., Nakano, K., Yamada, H., Matsuta, K., Han, H., Tokunaga, K., Ito, K., and Park, K.S. (1994). Association of complement C4 and HLA-DR alleles with systemic lupus erythematosus in Koreans. J. Rheumatol. *21*, 442–447.
24. Phelps, R.G., and Rees, A.J. (1999). The HLA complex in Goodpasture's disease: a model for analyzing susceptibility to autoimmunity. Kidney Int. *56*, 1638–1653.
25. Rubicz, R., Yolken, R., Drigalenko, E., Carless, M.A., Dyer, T.D., Bauman, L., Melton, P.E., Kent, J.W., Jr., Harley, J.B., Curran, J.E., et al. (2013). A genome-wide integrative genomic study localizes genetic factors influencing antibodies against Epstein-Barr virus nuclear antigen 1 (EBNA-1). PLoS Genet. *9*, e1003147.
26. Pender, M.P., and Burrows, S.R. (2014). Epstein-Barr virus and multiple sclerosis: potential opportunities for immunotherapy. Clin. Transl. Immunology *3*, e27.
27. Draborg, A.H., Duus, K., and Houen, G. (2013). Epstein-Barr virus in systemic autoimmune diseases. Clin. Dev. Immunol. *2013*, 535738.
28. Raychaudhuri, S., Sandor, C., Stahl, E.A., Freudenberg, J., Lee, H.S., Jia, X., Alfredsson, L., Padyukov, L., Klareskog, L., Worthington, J., et al. (2012). Five amino acids in three HLA proteins explain most of the association between MHC and seropositive rheumatoid arthritis. Nat. Genet. *44*, 291–296.
29. Han, B., Diogo, D., Eyre, S., Kallberg, H., Zhernakova, A., Bowes, J., Padyukov, L., Okada, Y., González-Gay, M.A., Rantapää-Dahlqvist, S., et al. (2014). Fine mapping seronegative and seropositive rheumatoid arthritis to shared and distinct HLA alleles by adjusting for the effects of heterogeneity. Am. J. Hum. Genet. *94*, 522–532.
30. Sundqvist, E., Buck, D., Warnke, C., Albrecht, E., Gieger, C., Khademi, M., Lima Bomfim, I., Fogdell-Hahn, A., Link, J., Alfredsson, L., et al.; International Multiple Sclerosis Genetics Consortium (2014). JC polyomavirus infection is strongly controlled by human leucocyte antigen class II variants. PLoS Pathog. *10*, e1004084.
31. Purcell, S., Cherny, S.S., and Sham, P.C. (2003). Genetic Power Calculator: design of linkage and association genetic mapping studies of complex traits. Bioinformatics *19*, 149–150.

The American Journal of Human Genetics
Supplemental Data

Amino Acid Variation in HLA Class II Proteins Is a Major Determinant of Humoral Response to Common Viruses

Christian Hammer, Martin Begemann, Paul J. McLaren, István Bartha, Angelika Michel, Beate Klose, Corinna Schmitt, Tim Waterboer, Michael Pawlita, Thomas F. Schulz, Hannelore Ehrenreich, and Jacques Fellay

Supplemental Figures.

Figure S1. Principal component analysis.

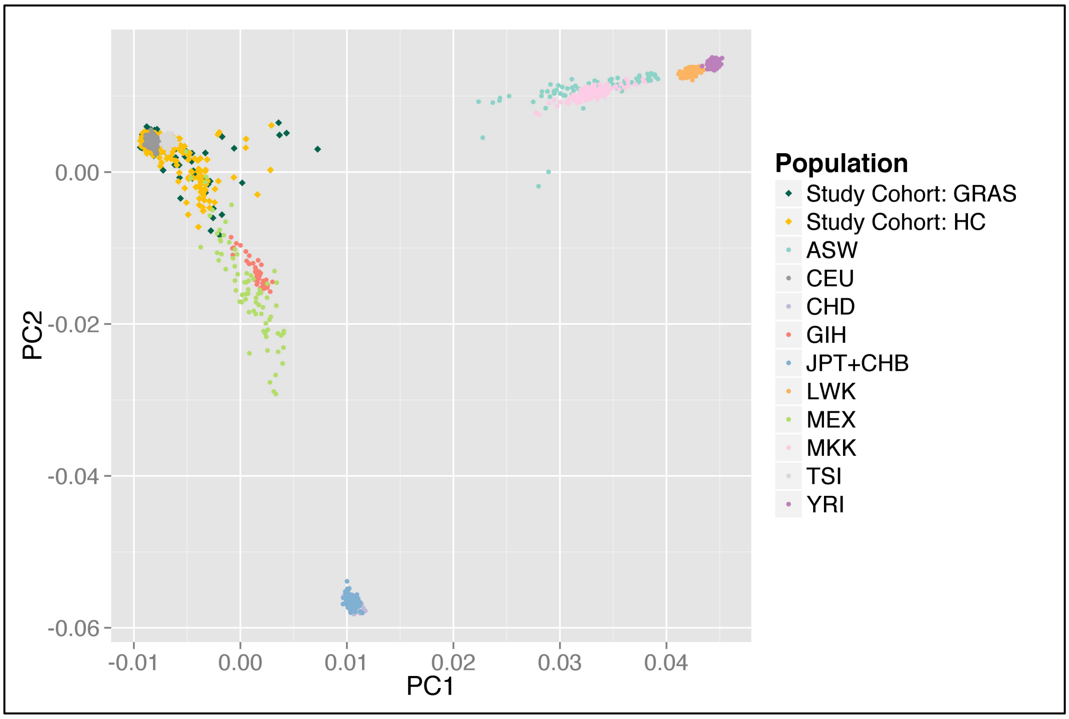

Study participants are displayed together with Hapmap version3 r2 samples, according to their first two principal components (PC1, PC2) as generated using GCTA (v1.24). GRAS, Göttingen Research Association for Schizophrenia patients; HC, Neuropsychiatrically healthy controls; ASW, African ancestry in Southwest USA; CEU, Utah residents with Northern and Western European ancestry from the CEPH collection; CHB, Han Chinese in Beijing, China; CHD, Chinese in Metropolitan Denver, Colorado; GIH, Gujarati Indians in Houston, Texas; JPT, Japanese in Tokyo, Japan; LWK, Luhya in Webuye, Kenya; MXL, Mexican Ancestry in Los Angeles, California; MKK, Maasai in Kinyawa, Kenya, TSI, Toscani in Italia; YRI, Yoruba in Ibadan, Nigeria.

Figure S2. Quantile-quantile plot for assessment of test statistic inflation.

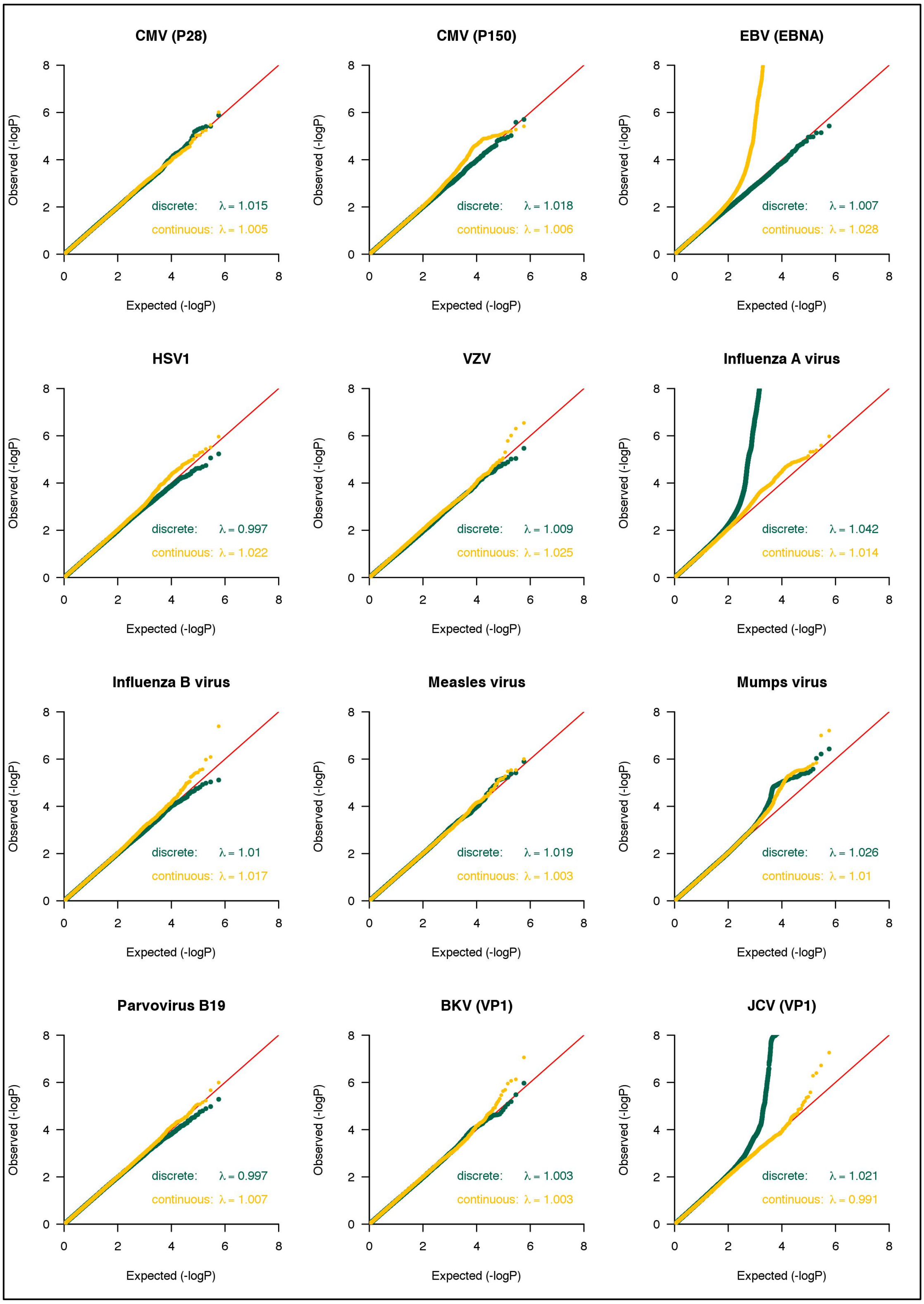

Continued on next page.

Figure S2. Quantile-quantile plot for assessment of test statistic inflation (continued).

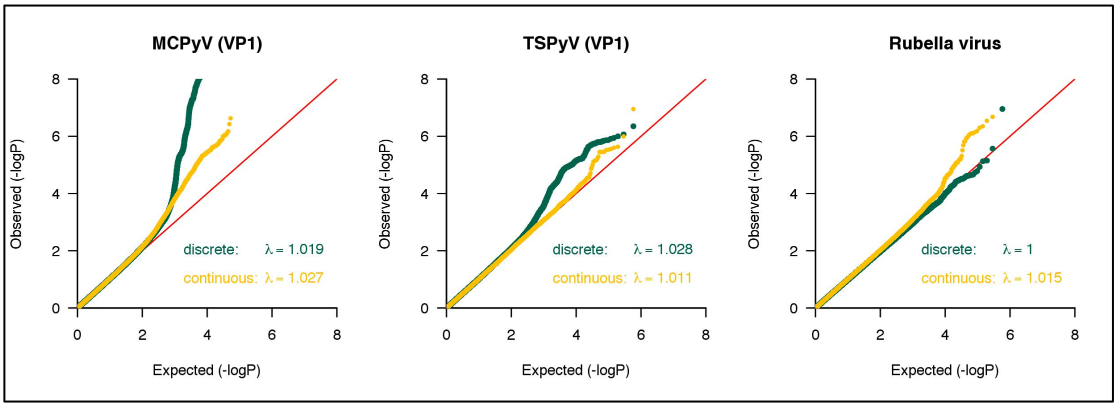

Quantile-quantile plot for each virus, including results for serostatus (discrete trait, green) and IgG levels (continuous trait, yellow). For each tested variant, the –log10 *P* value is plotted against the null distribution (red line). λ, genomic inflation factor.

Figure S3. Manhattan plots of association results.

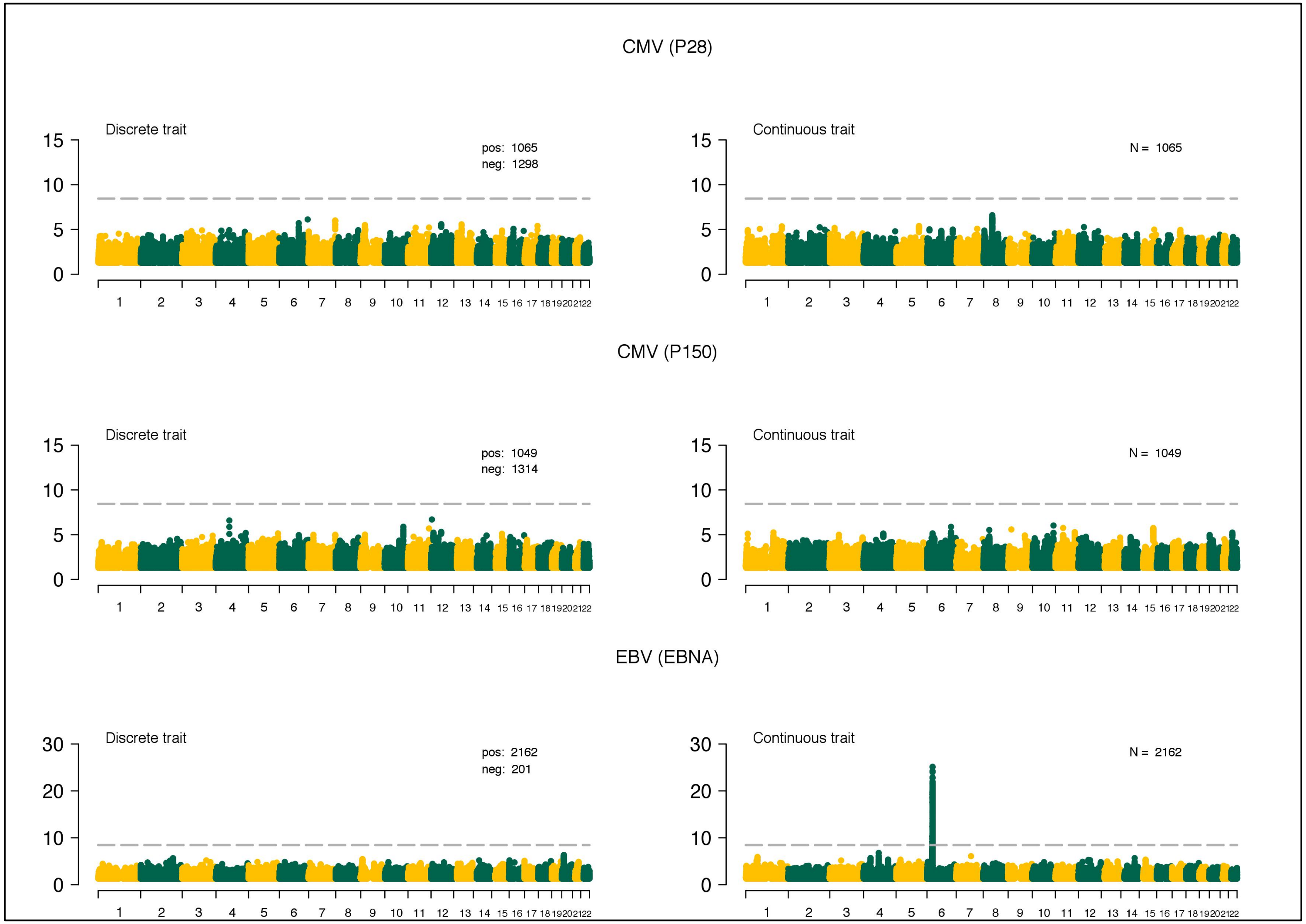

Continued on next page.

Figure S3. Manhattan plots of association results (continued).

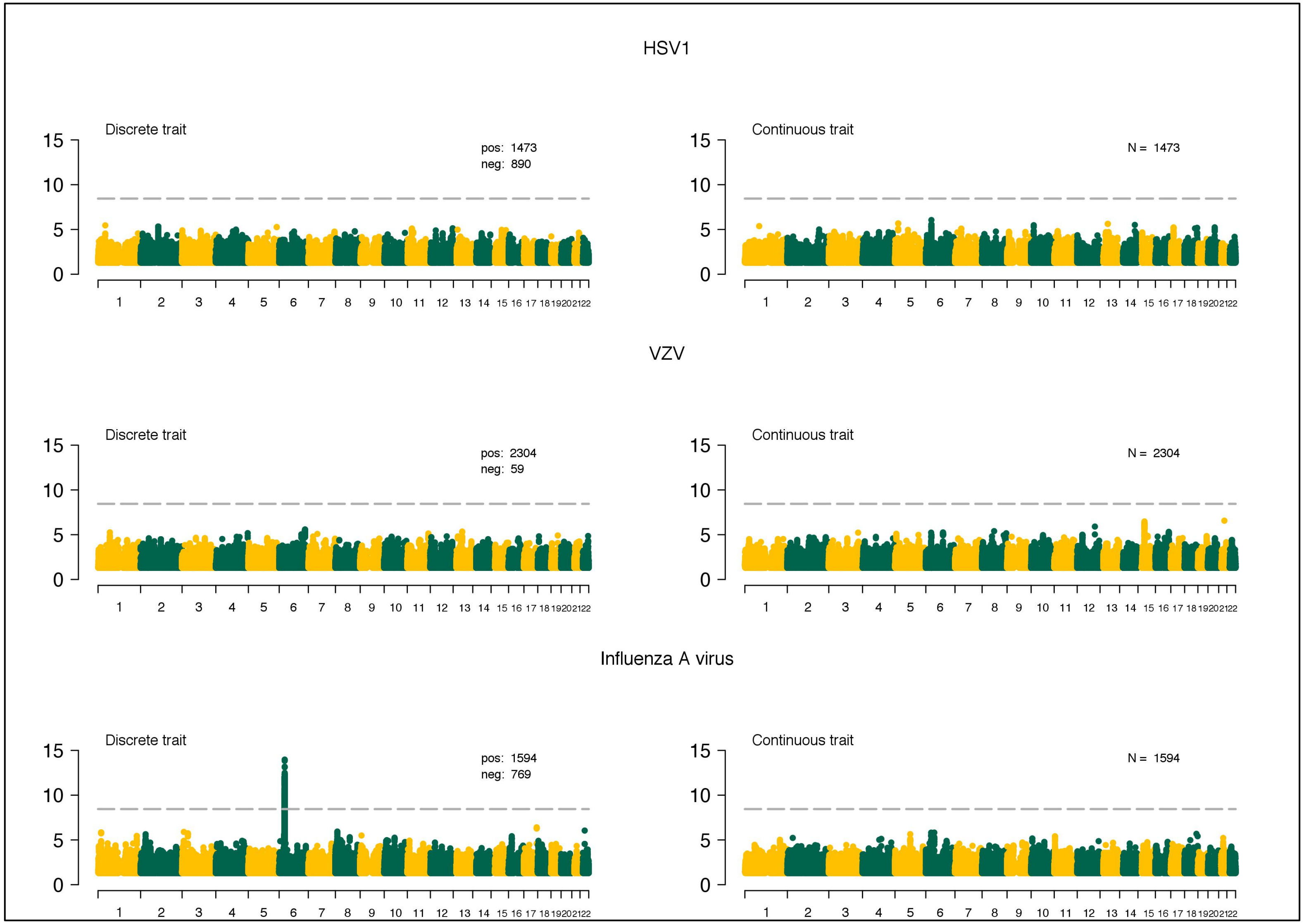

Continued on next page.

Figure S3. Manhattan plots of association results (continued).

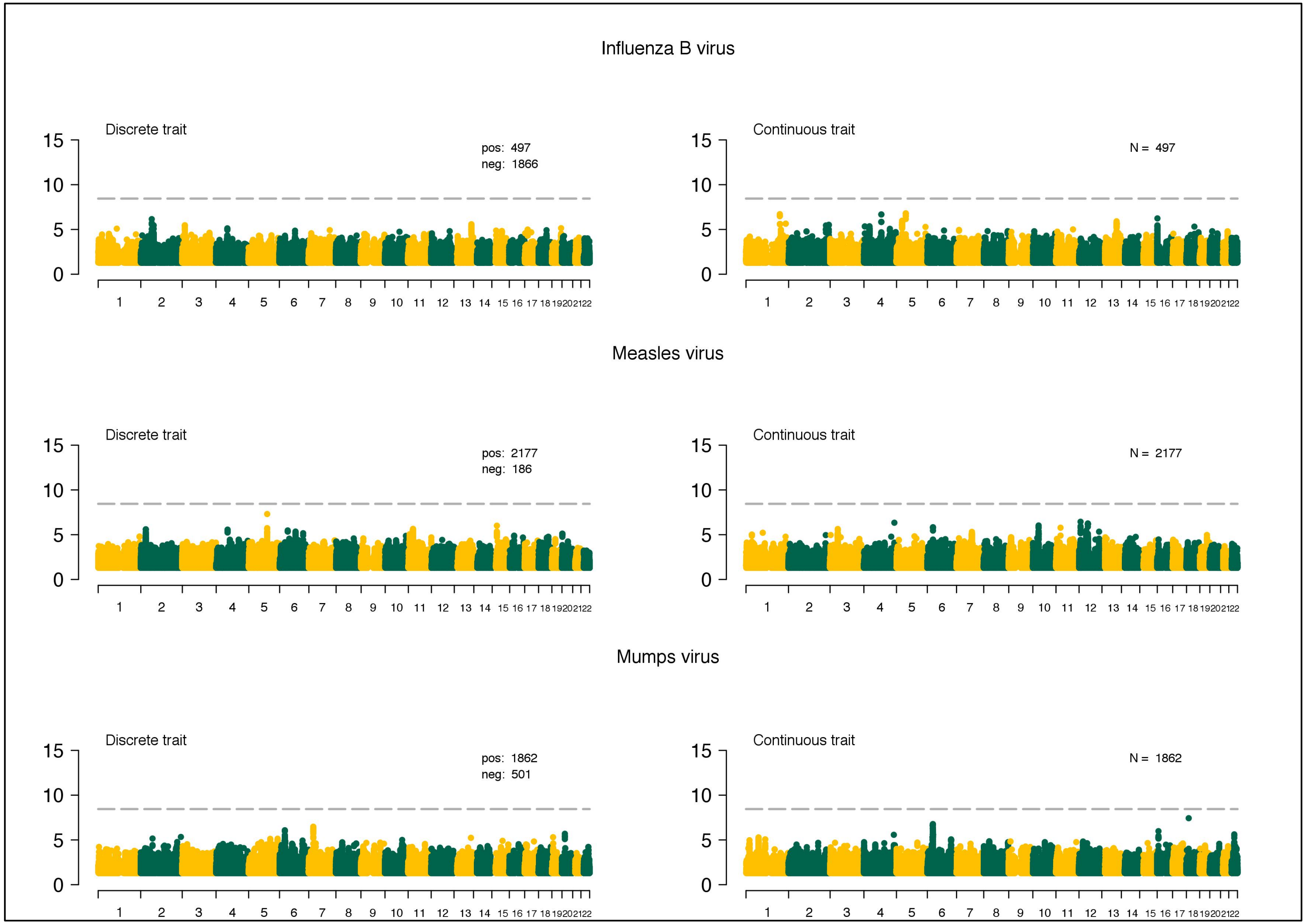

Continued on next page.

Figure S3. Manhattan plots of association results (continued).

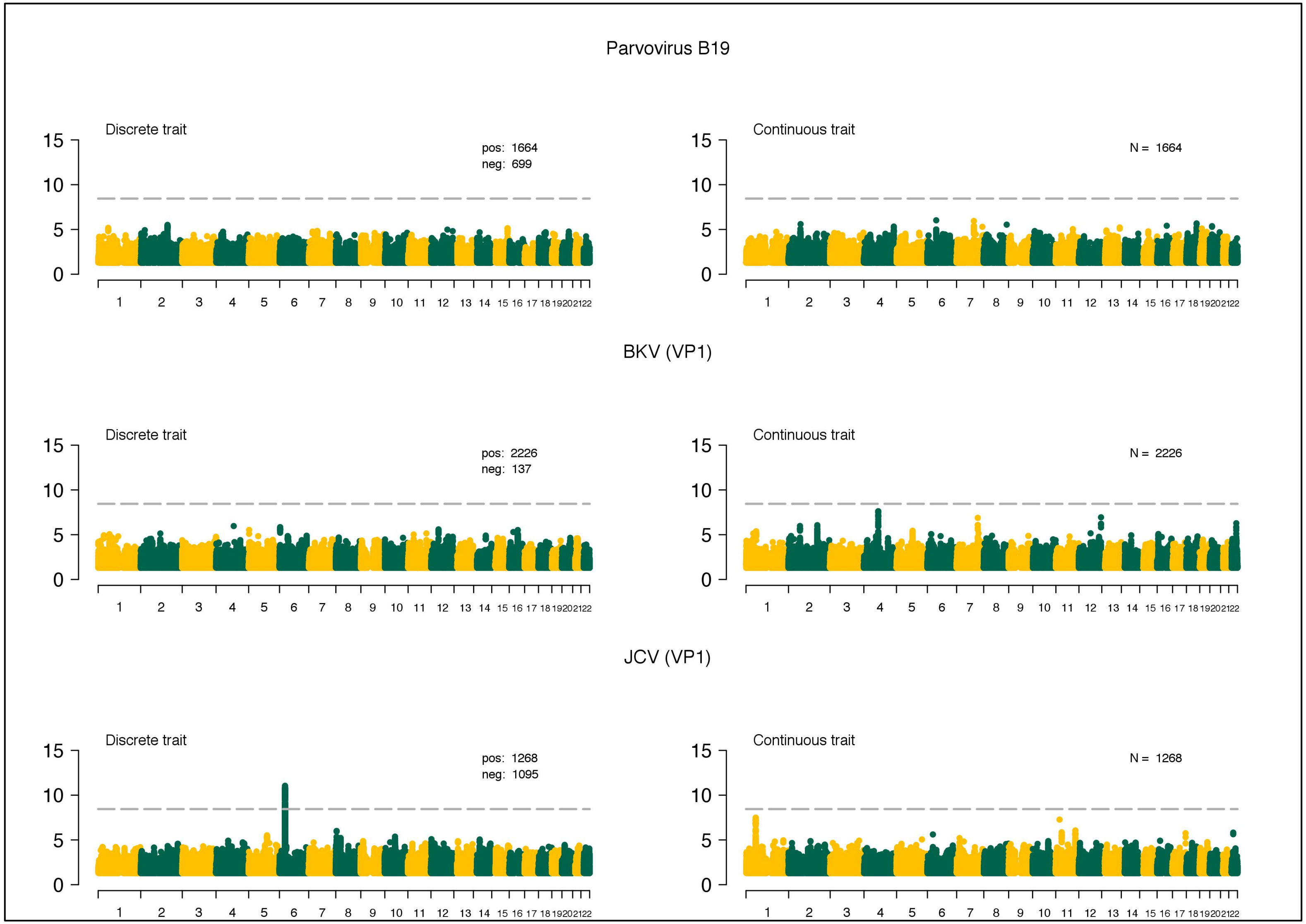

Continued on next page.

Figure S3. Manhattan plots of association results (continued).

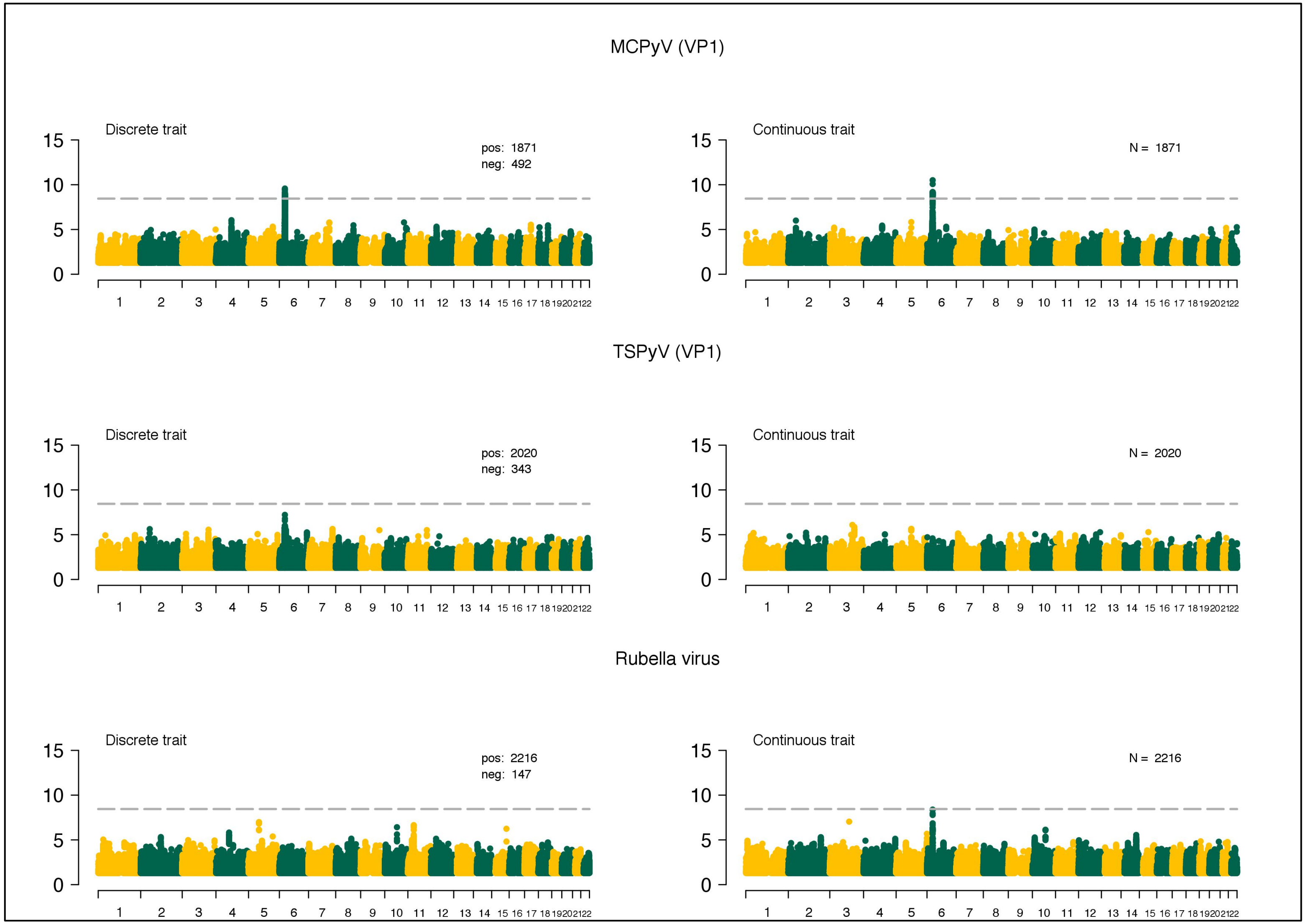

Manhattan plots of the association results for each tested virus, both for serostatus (discrete trait, left panel) and IgG levels (continuous trait, right panel). The number of included subjects is given for each analysis. Each variant is plotted by genomic position (x-axis) and -log10(*P*) value (y-axis). The dashed horizontal line denotes genome-wide significance (*P* = 3.57 x 10-9).

Figure S4. GWAS and HLA association results for EBV anti-EBNA IgG levels.

A

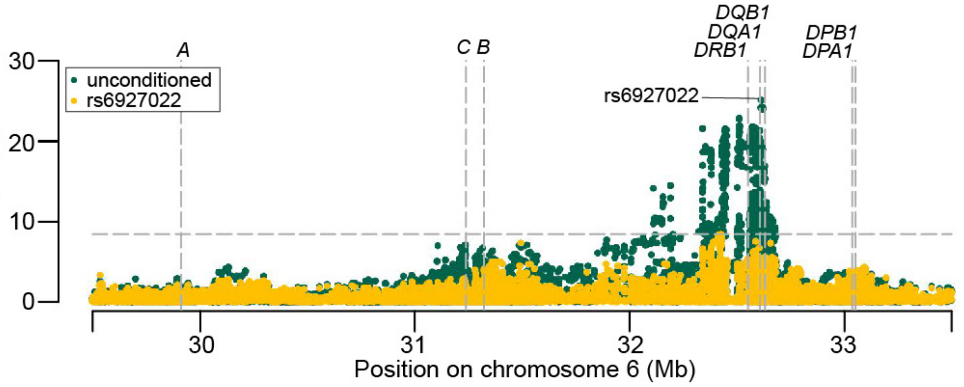

B

HLA-DRβ1 (11)	*HLA-DRB1* alleles	frequency	Beta (95% CI)
Leu	*01:01	11.1%	-0.02 (-0.06,0.03)
Ser	*03:01,*08:01,*11:01,*11:04,*12:01,*13:01, *13:02,*13:03,*14:01	42.9%	0.08 (0.06,0.11)
Val	*04:01,*04:04	15.7%	-0.10 (-0.14,-0.06)
Gly	*07:01	12.0%	-0.17 (-0.21,-0.12)
Pro	*15:01,*16:01	17.4%	0.07 (0.04,0.11)
**HLA-DRβ1 (26)***	***HLA-DRB1* alleles**	**frequency**	**Beta (95% CI)**
Leu	*01:01,*12:01	14.1%	0.10 (0.02,0.18)
Tyr	*03:01	11.6%	-0.16 (-0.21,-0.10)
Phe	*04:01,*04:04,*07:01,*08:01,*11:01,*11:04, *13:01,*13:02,*13:03,*14:01,*15:01,*16:01	74.3%	0.09 (0.04,0.13)

*, conditioning on HLA-DRβ1 (11)

C

Covariates	Risk allele	P	Beta (95% CI)
age, sex, PC1-3	*HLA-DRB1*07:01*	1.0×10^{-14}	-0.166 (-0.21,-0.12)
+ *HLA-DRB1*07:01*	*HLA-DRB1*03:01*	2.6×10^{-9}	-0.115 (-0.15,-0.08)
+ *HLA-DRB1*03:01*	*HLA-DRB1*04:04*	1.2×10^{-5}	-0.207 (-0.30,-0.12)

(A) Regional association plot of GWAS results for EBV anti-EBNA IgG levels of 2162 seropositive individuals. Values of –log10(*P*) are plotted by their position in the MHC genomic region on chromosome 6. The most significant association was observed for rs6927022 ($P = 7.3 \times 10^{-26}$, beta = 0.16 for the A allele). Accounting for the effect of rs6927022, we observed no further independent association. The dashed horizontal line indicates the threshold for genome-wide significance. The annotated dashed vertical lines indicate the positions of the classical HLA genes. (B) Effect estimates for amino acid residues at position 11 (omnibus $P = 5.9 \times 10^{-23}$) and 26 (conditional omnibus $P = 2.5 \times 10^{-9}$) of HLA-DRβ1. Designated classical HLA alleles contain the respective amino acid residue at the given position. CI, confidence interval. (C) Association results and conditional regression for classical HLA alleles. Conditioning on *HLA-DRB1*07:01* revealed *HLA-DRB1*03:01* to be independently associated. PC, principal component; CI, confidence interval.

Figure S5. GWAS and HLA association results for JCPyV serostatus.

A

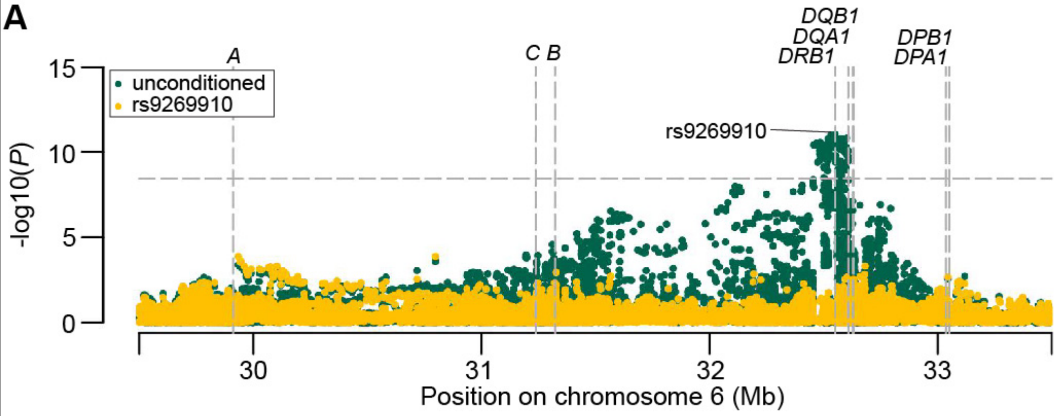

B

HLA-DRβ1 (133)	*HLA-DRB1* alleles	frequency	OR (95% CI)
Arg	*01:01,*03:01,*04:01,*04:04,*07:01,*08:01, *11:01,*11:03,*11:04,*12:01,*13:01,*13:02, *13:03,*14:01	82.6%	1.71 (1.46,2.01)
Leu	*15:01,*16:01	17.4%	0.58 (0.50,0.68)

C

Covariates	Risk allele	P	OR (95% CI)
age, sex, PC1-3, diagnosis	*HLA-DQA1*01:02*	4.1×10^{-9}	0.64 (0.56,0.75)
+ *HLA-DQA1*01:02*	*HLA-DRB1*04:04*	2.8×10^{-4}	0.46 (0.31,0.70)

(A) Regional association plot of GWAS results for JCPyV serostatus, comparing 1268 seropositive and 1095 seronegative individuals. Values of –log10(*P*) are plotted by their position in the MHC genomic region on chromosome 6. The most significant association was observed for rs9269910 ($P = 8.9 \times 10^{-12}$, OR = 1.74 for the A allele). Accounting for the effect of rs9269910, we observed no further independent association. The dashed horizontal line indicates the threshold for genome-wide significance. The annotated dashed vertical lines indicate the positions of the classical HLA genes. (B) Effect estimates for amino acid residues at position 133 of HLA-DRβ1 (omnibus $P = 1.1 \times 10^{-11}$). Designated classical HLA alleles contain the respective amino acid residue at the given position. OR, odds ratio; CI, confidence interval. (C) Association results and conditional regression for classical HLA alleles. Conditioning on *HLA-DQA1*01:02* did not reveal further independent associations (threshold: P = 3.57×10^{-09}). PC, principal component; OR, odds ratio; CI, confidence interval.

Figure S6. GWAS and HLA association results for MCPyV serostatus.

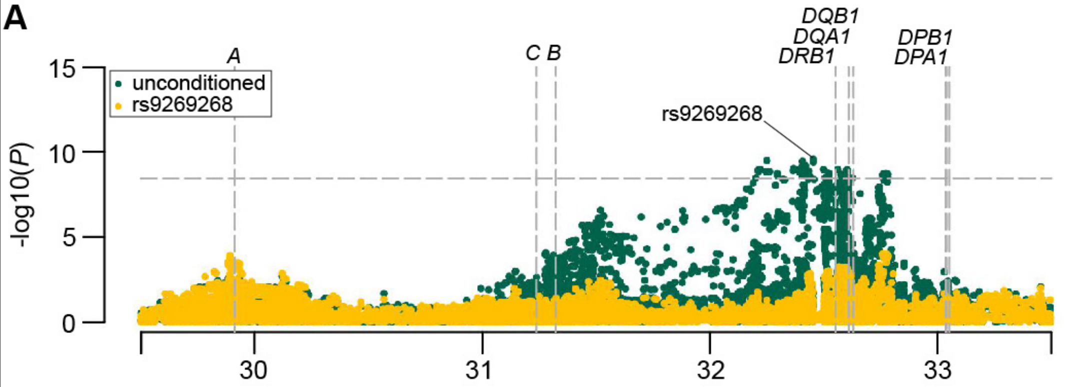

B

HLA-DRβ1 (13)	*HLA-DRB1* alleles	frequency	OR (95% CI)
Phe	*01:01	12.6%	1.42 (1.13,1.78)
Ser	*03:01,*11:01,*11:03,*11:04,*13:01,*13:02, *13:03,*14:01	37.7%	1.45 (1.24,1.69)
His	*04:01,*04:04	15.0%	0.81 (0.67,0.97)
Tyr	*07:01	12.0%	0.98 (0.79,1.21)
Gly	*08:01,*12:01	5.3%	0.89 (0.66,1.20)
Arg	*15:01,*16:01	17.4%	0.60 (0.50,0.71)

C

Covariates	Risk allele	P	OR (95% CI)
age, sex, PC1-3	*HLA-DQB1*06:02*	1.4×10^{-9}	0.56 (0.46,0.67)
+ *HLA-DQB1*06:02*	*HLA-DQA1*03:01*	1.0×10^{-3}	0.73 (0.60,0.88)

(A) Regional association plot of GWAS results for MCPyV serostatus, comparing 1871 seropositive and 492 seronegative individuals. Values of –log10(*P*) are plotted by their position in the MHC genomic region on chromosome 6. The most significant association was observed for rs9269268 ($P = 2.7 \times 10^{-10}$, OR = 1.53 for the C allele). Accounting for the effect of rs9269268, we observed no further independent association. The dashed horizontal line indicates the threshold for genome-wide significance. The annotated dashed vertical lines indicate the positions of the classical HLA genes. (B) Effect estimates for amino acid residues at position 13 of HLA-DRβ1 (omnibus $P = 1.9 \times 10^{-10}$). Designated classical HLA alleles contain the respective amino acid residue at the given position. OR, odds ratio; CI, confidence interval. (C) Association results and conditional regression for classical HLA alleles. Conditioning on *HLA-DQB1*06:02* did not reveal further independent associations (threshold: P = 3.57×10^{-09}). PC, principal component; OR, odds ratio; CI, confidence interval.

Figure S7. GWAS association results for MCPyV IgG levels.

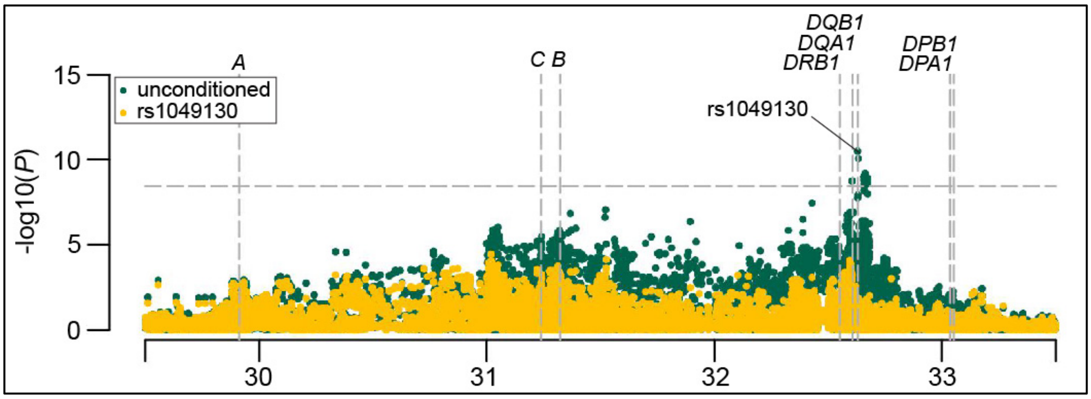

Regional association plot of GWAS results for MCPyV IgG levels of 1871 seropositive individuals. Values of –log10(*P*) are plotted by their position in the MHC genomic region on chromosome 6. The most significant association was observed for rs1049130 (P = 3.2 x 10^{-11}, beta = 0.22 for the G allele). Accounting for the effect of rs1049130, we observed no further independent association. The dashed horizontal line indicates the threshold for genome-wide significance. The annotated dashed vertical lines indicate the positions of the classical HLA genes.

Supplemental Tables.

Table S1. Description of serological assays.

Virus	Test / Epitope	Details of Antigens for Serology Testing
**CMV**[1]	P28	Strain “Town” (FJ616285), small nucleocapsid protein p28
	P150	Strain “Town” (FJ616285), N-terminal part of tegument protein p150, amino acids 1-550
**EBV**[1; 2]	EBNA	Strain “B-95-8” (P03211.1), truncated protein: C-terminal part, amino acids 325-641
**HSV1**	Novagnost HSV1 IgG	Commercially available purified recombinant glycoprotein G, expressed in eukaryotic cells (Siemens Novagnost)
**VZV**	Enzygnost Anti-VZV / IgG	Commercially available antigen, strain “Ellen”, prepared from infected human diploid lung fibroblasts (Siemens Enzygnost)
**Influenza A**	Novagnost Influenza A IgG	Commercially available cell-free H3N2 virus preparation, cultured in chicken eggs (Siemens Novagnost)
**Influenza B**	Novagnost Influenza B IgG	Commercially available Influenza B strain, cultured in eukaryotic cells (Siemens Novagnost)
**Measles**	Enzygnost Anti-Measles Virus / IgG	Commercially available antigen, strain “Edmonston”, prepared from infected Vero cells (Siemens Enzygnost)
**Mumps**	Enzygnost Anti-Parotitis Virus / IgG	Commercially available antigen, strain “Enders”, prepared from infected human Vero cells (Siemens Enzygnost)
**Parvo B19**	Novagnost Parvovirus B 19 IgG	Commercially available purified recombinant VLP antigen, expressed in yeast (Siemens Novagnost)
**BKPyV**[3; 4]	VP1	Strain “AS” (P14996.1), major capsid protein VP1
**JCPyV**[3; 4]	VP1	Strain “GS/B” (NC_001699.1), major capsid protein VP1
**MCPyV**[5; 6]	VP1	Isolate “344” (JF812999.1), major capsid protein VP1
**TSPyV**[7]	VP1	(NC_014361.1), major capsid protein VP1
**Rubella**	Enzygnost Anti-Rubella Virus / IgG	Commercially available antigen, strain RA27/3, substrain B1272, prepared from infected BHK cells (Siemens Enzygnost)

NCBI accession numbers are given in brackets, where available.

Table S2. Impact of age and sex on serostatus and IgG levels.

	Age				Sex			
	Serostatus		IgG levels		Serostatus		IgG levels	
**Virus**	***P*** **(logistic regression)**	**OR (95% CI)**	***P*** **(linear regression)**	**Beta (95% CI)**	***P*** **(logistic regression)**	**OR (reference = male) (95% CI)**	***P*** **(linear regression)**	**Beta (reference = male) (95% CI)**
**BKPyV**	**3.0E-03**	0.982 (0.970,0.994)	**1.9E-26**	-0.016 (-0.019,-0.013)	5.9E-01	0.907 (0.636,1.292)	8.4E-01	-0.009 (-0.093,0.076)
**CMV PP150**	**2.0E-16**	1.026 (1.020,1.033)	7.0E-03	0.006 (0.002,0.011)	1.2E-01	1.141 (0.964,1.350)	**2.3E-07**	0.339 (0.211,0.466)
**CMV PP28**	**2.9E-12**	1.022 (1.016,1.028)	8.0E-03	0.006 (0.002,0.011)	4.8E-02	1.185 (1.002,1.402)	**2.0E-03**	0.211 (0.077,0.346)
**EBV**	**1.2E-09**	1.039 (1.026,1.052)	7.0E-01	0.000 (-0.002,0.001)	5.0E-03	1.584 (1.148,2.186)	8.5E-02	-0.036 (-0.076,0.005)
**HSV1**	**1.3E-30**	1.041 (1.034-1.048)	4.5E-02	0.002 (0.000-0.003)	1.9E-01	1.125 (0.945,1.338)	5.8E-02	-0.040 (-0.082,0.001)
**Influenza A**	2.3E-01	0.996 (0.990,1.002)	**5.4E-05**	0.003 (0.001,0.004)	4.7E-01	1.068 (0.892,1.277)	2.6E-01	0.028 (-0.017,0.062)
**Influenza B**	**2.0E-17**	1.032 (1.025,1.040)	9.0E-03	-0.002 (-0.003,0.000)	4.1E-02	0.804 (0.652,0.991)	9.9E-02	-0.027 (-0.060,0.005)
**JCPyV**	**1.6E-10**	1.020 (1.014,1.027)	3.5E-01	0.002 (-0.002,0.005)	3.2E-02	0.832 (0.703,0.984)	1.5E-02	-0.117 (-0.211,-0.022)
**MCPyV**	**1.7E-04**	1.015 (1.007,1.023)	**1.2E-04**	0.007 (0.003,0.010)	1.1E-01	0.846 (0.690,1.037)	7.8E-01	-0.014 (-0.111,0.083)
**Measles**	**2.5E-22**	1.084 (1.066,1.102)	**1.1E-111**	0.031 (0.029,0.034)	2.1E-01	1.227 (0.892,1.689)	9.0E-03	0.106 (0.026,0.186)
**Mumps**	**1.0E-19**	1.039 (1.031,1.048)	**3.5E-04**	0.004 (0.002,0.007)	3.7E-02	1.249 (1.013,1.540)	**4.1E-04**	0.123 (0.055,0.192)
**Parvo**	2.6E-01	0.996 (0.990,1.003)	**4.8E-12**	-0.005 (-0.006,-0.004)	7.0E-03	0.780 (0.650,0.935)	**3.0E-06**	0.093 (0.054,0.132)
**Rubella**	**6.0E-10**	1.049 (1.033,1.065)	1.5E-01	-0.002 (-0.005,0.001)	**5.6E-08**	3.677 (2.298,5.882)	**9.0E-07**	-0.188 (-0.262,-0.113)
**TSPyV**	**3.1E-08**	1.026 (1.017,1.036)	**1.7E-07**	-0.010 (-0.013,-0.006)	1.2E-01	0.832 (0.658,1.051)	**2.0E-03**	-0.160 (-0.262,-0.059)
**VZV**	1.1E-01	1.017 (0.996,1.037)	1.1E-01	-0.002 (-0.003,0.000)	4.9E-01	1.217 (0.700,2.115)	4.1E-01	0.022 (-0.031,0.076)

Significant *P* values (corrected for the number of viruses) are displayed in bold. OR, odds ratio; CI, confidence interval.

Table S3. Comparison of serostatus and IgG levels between psychiatric disease patients and healthy control individuals.

	Serostatus				IgG levels			
	N seropositive (%)				Mean IgG (SE)			
Virus	Healthy controls	Psychiatric diagnosis	*P* (logistic regression)	OR (95% CI)	Healthy controls	Psychiatric diagnosis	*P* linear regression)	Beta (95% CI)
**BKPyV**	1090 (95.0)	1136 (93.4)	1.4E-01	0.76 (0.53,1.09)	8.40 (0.03)	8.49 (0.03)	**2.1E-04**	0.15 (0.07,0.23)
**CMV PP150**	518 (45.2)	531 (43.7)	**2.0E-03**	0.76 (0.64,0.90)	7.72 (0.04)	7.99 (0.05)	**2.0E-04**	0.24 (0.11,0.36)
**CMV PP28**	522 (45.5)	543 (44.7)	1.2E-02	0.80 (0.68,0.95)	7.90 (0.05)	8.04 (0.05)	1.8E-01	0.09 (-0.04,0.22)
**EBV**	1071 (93.4)	1091 (89.7)	**4.9E-05**	0.53 (0.39,0.72)	8.86 (0.01)	8.92 (0.01)	1.0E-02	0.05 (0.01,0.09)
**HSV1**	699 (60.9)	774 (63.7)	5.4E-01	0.95 (0.79,1.13)	10.35 (0.01)	10.42 (0.01)	**2.0E-03**	0.07 (0.03,0.11)
**Influenza A**	774 (67.5)	820 (67.4)	8.0E-01	1.02 (0.86,1.22)	9.97 (0.01)	9.94 (0.01)	5.2E-02	-0.04 (-0.08,0.00)
**Influenza B**	236 (20.6)	261 (21.5)	5.0E-01	0.93 (0.76,1.15)	9.51 (0.01)	9.55 (0.01)	**1.0E-03**	0.05 (0.02,0.08)
**JCPyV**	546 (47.6)	722 (59.4)	**7.0E-06**	1.47 (1.24,1.73)	7.11 (0.03)	7.17 (0.03)	2.7E-01	0.05 (-0.04,0.14)
**MCPyV**	893 (77.9)	978 (80.4)	6.7E-01	1.05 (0.85,1.28)	7.97 (0.04)	8.04 (0.03)	4.5E-01	0.04 (-0.06,0.13)
**Measles**	1044 (91.0)	1133 (93.2)	9.1E-01	1.02 (0.74,1.40)	14.99 (0.03)	15.14 (0.03)	1.0E-01	0.06 (-0.01,0.13)
**Mumps**	902 (78.6)	960 (78.9)	3.0E-01	0.90 (0.73,1.10)	14.53 (0.02)	14.62 (0.02)	2.6E-02	0.08 (0.01,0.14)
**Parvo**	849 (74.0)	815 (67.0)	**2.2E-04**	0.71 (0.59,0.85)	10.91 (0.01)	10.85 (0.01)	2.0E-02	-0.04 (-0.08.-0.01)
**Rubella**	1089 (94.9)	1127 (92.7)	6.0E-03	0.61 (0.43,0.87)	11.41 (0.03)	11.41 (0.03)	8.4E-01	-0.01 (-0.08,0.07)
**TSPyV**	973 (84.8)	1047 (86.1)	9.4E-01	0.99 (0.78,1.26)	8.91 (0.03)	8.95 (0.04)	6.8E-02	0.09 (-0.01,0.19)
**VZV**	1128 (98.3)	1176 (96.7)	7.0E-03	0.46 (0.26,0.81)	13.72 (0.02)	13.78 (0.02)	7.0E-03	0.07 (0.02,0.12)

Age, sex, and the first three principal components are included as covariates in all regression analyses. Significant *P* values (corrected for the number of viruses) are displayed in bold. OR, odds ratio; CI, confidence interval; SE, standard error of the mean.

Table S6. Residual significance of GWAS SNPs conditioning for the associated HLA alleles and variable amino acids.

Virus	Phenotype	Covariates	SNP	*P* (SNP)	Associated classical HLA alleles	*P* (SNP) conditional on significant HLA alleles
**EBV**	IgG levels	age,sex,PC1-3	rs6927022	7.35×10^{-26}	DRB1*07:01, DRB1*03:01	2.59×10^{-11}
**Influenza A**	Serostatus	age,sex,PC1-3	rs140012631	1.06×10^{-14}	DQB1*05:01	4.47×10^{-4}
**JCPyV**	Serostatus	age,sex,PC1-3,diagnosis	rs9269910	8.88×10^{-12}	DQA1*01:02	3.63×10^{-4}
**MCPyV**	Serostatus	age,sex,PC1-3	rs9269268	2.67×10^{-10}	DQB1*06:02	2.74×10^{-2}
**Virus**	**Phenotype**	**Covariates**	**SNP**	***P* (SNP)**	**Associated HLA- DRβ1 amino acid positions**	***P* (SNP) conditional on significant amino acids**
**EBV**	IgG levels	age,sex,PC1-3	rs6927022	7.35×10^{-26}	11, 26	4.20×10^{-2}
**Influenza A**	Serostatus	age,sex,PC1-3	rs140012631	1.06×10^{-14}	96	9.48×10^{-4}
**JCPyV**	Serostatus	age,sex,PC1-3,diagnosis	rs9269910	8.88×10^{-12}	133	1
**MCPyV**	Serostatus	age,sex,PC1-3	rs9269268	2.67×10^{-10}	13	4.34×10^{-2}

PC, principal component.

Table S9. Variance explained by the associated variable HLA amino acids.

Study Design	Virus	Nagelkerke's pseudo-r^2 (age, sex, PC1-3)	associated amino acid positions	Nagelkerke's pseudo-r^2 (age, sex, PC1-3, amino acids)	Δr^2
Discrete	Influenza A	0.4%	HLA-DRβ1 (96)	4.8%	4.4%
	JCPyV[a]	4.7%	HLA-DRβ1 (133)	7.2%	2.5%
	MCPyV	2.4%	HLA-DRβ1 (13)	5.9%	3.5%
**Study Design**	**Virus**	**r^2 (age, sex, PC1-3)**	**associated amino acid positions**	**r^2 (age, sex, PC1-3, amino acids)**	**Δr^2**
Continuous	EBV	1.2%	HLA-DRβ1 (11,26)	6.6%	5.4%

[a],additionally corrected for diagnosis (psychiatric patients vs. healthy controls)

Table S10. Correlations of effect estimates between analyses of serostatus and IgG levels.

Virus	N seropositive (%)	Variable HLA amino acid positions	
		Pearson's r	*P*
**EBV**	2162 (91.5)	0.39	< 2.2E-16
**Influenza A**	1594 (67.5)	0.62	< 2.2E-16
**JCPyV**	1268 (53.7)	0.66	< 2.2E-16
**MCPyV**	1871 (79.2)	0.55	< 2.2E-16

Correlation analyses between odds ratios of logistic regression analyses on serostatus and betas of linear regression analyses on IgG levels, taking into account all variable amino acid positions. r, correlation coefficient

Supplemental References.

1. Brozy, J. (2009). Development of Multiplex Serology for HSV-1, HSV-2, EBV and HCMV using Recombinant Proteins. Ruprecht-Karls-Universität Heidelberg, Heidelberg.
2. Teras, L.R., Rollison, D.E., Pawlita, M., Michel, A., Brozy, J., de Sanjose, S., Blase, J.L., and Gapstur, S.M. (2015). Epstein-Barr virus and risk of non-Hodgkin lymphoma in the cancer prevention study-II and a meta-analysis of serologic studies. International journal of cancer Journal international du cancer 136, 108-116.
3. Antonsson, A., Green, A.C., Mallitt, K.A., O'Rourke, P.K., Pawlita, M., Waterboer, T., and Neale, R.E. (2010). Prevalence and stability of antibodies to the BK and JC polyomaviruses: a long-term longitudinal study of Australians. The Journal of general virology 91, 1849-1853.
4. Kjaerheim, K., Roe, O.D., Waterboer, T., Sehr, P., Rizk, R., Dai, H.Y., Sandeck, H., Larsson, E., Andersen, A., Boffetta, P., et al. (2007). Absence of SV40 antibodies or DNA fragments in prediagnostic mesothelioma serum samples. International journal of cancer Journal international du cancer 120, 2459-2465.
5. Robles, C., Casabonne, D., Benavente, Y., Costas, L., Gonzalez-Barca, E., Aymerich, M., Campo, E., Tardon, A., Jimenez-Moleon, J.J., Castano-Vinyals, G., et al. (2015). Seroreactivity against Merkel cell polyomavirus and other polyomaviruses in chronic lymphocytic leukemia, the MCC-Spain study. The Journal of general virology.
6. Rollison, D.E., Giuliano, A.R., Messina, J.L., Fenske, N.A., Cherpelis, B.S., Sondak, V.K., Roetzheim, R.G., Iannacone, M.R., Michael, K.M., Gheit, T., et al. (2012). Case-control study of Merkel cell polyomavirus infection and cutaneous squamous cell carcinoma. Cancer epidemiology, biomarkers & prevention : a publication of the American Association for Cancer Research, cosponsored by the American Society of Preventive Oncology 21, 74-81.
7. Teras, L.R., Rollison, D.E., Pawlita, M., Michel, A., Blase, J.L., Willhauck-Fleckenstein, M., and Gapstur, S.M. (2015). Prediagnostic circulating polyomavirus antibody levels and risk of non-Hodgkin lymphoma. Cancer epidemiology, biomarkers & prevention : a publication of the American Association for Cancer Research, cosponsored by the American Society of Preventive Oncology 24, 477-480.
